# Supplementary material for: Genomic Landscape Highlights Molecular Mechanisms Involved in Silicate Solubilization, Stress Tolerance, and Potential Growth-Promoting Activity of Bacterium Enterobacter sp. LR6
Source: Cells. 2022 Nov 15;11(22):3622. doi: 10.3390/cells11223622 (PMC9688052; doi:10.3390/cells11223622)
Supplement: Supplementary file 1 [file cells-11-03622-s001.zip › cells-1917087-supplementary/cells-1917087-revised SM/Supplementary figures.pdf]

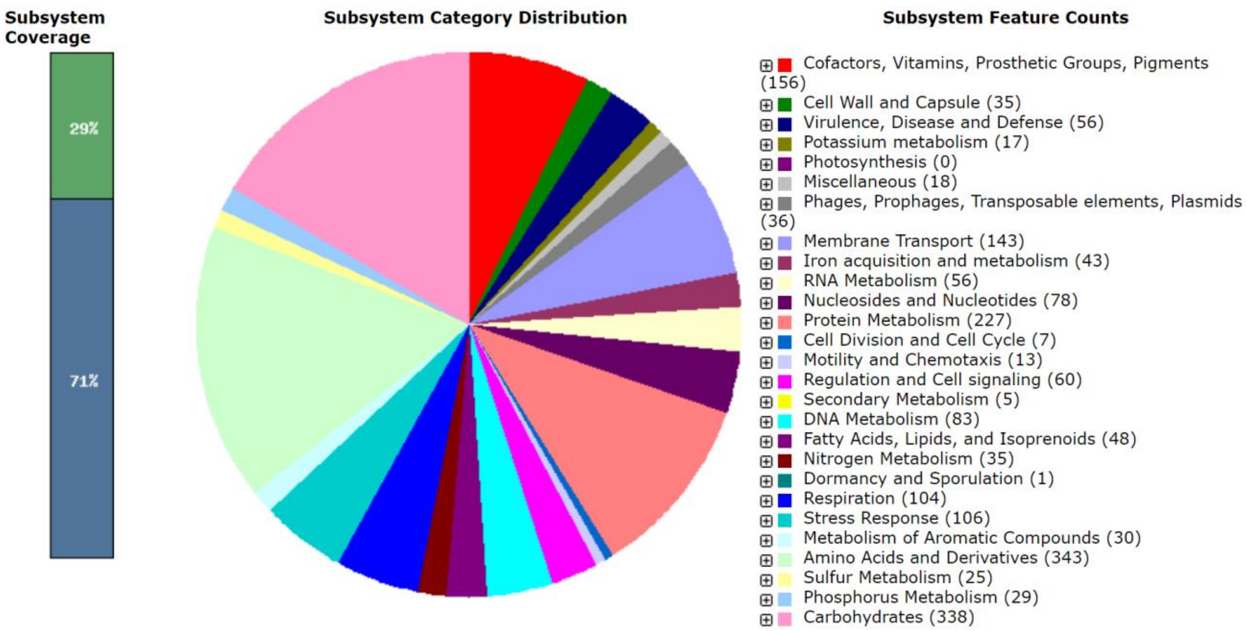


**Figure S1.** Subsystem distribution and feature counts of various category in *Enterobacter sp.* LR6 genome

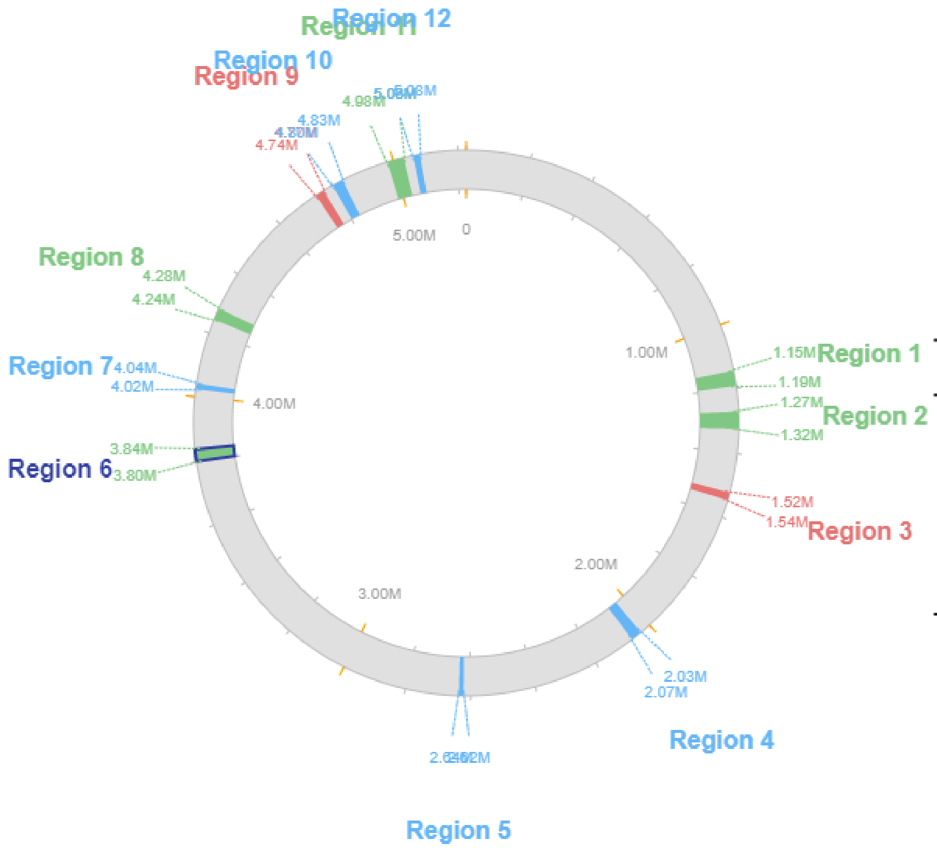


| Features | |
|---|---|
| Length | 5226212 |
| GC% | 51.8 |
| Prophage region | 6 |
| Phages | 12 |

**Figure S2.** The view of detected prophage area in the genome of *Enterobacter sp.* LR6 by using PHASTER tool. The green region on genome map represent the complete, blue relatively complete and red represent the incomplete prophage area.

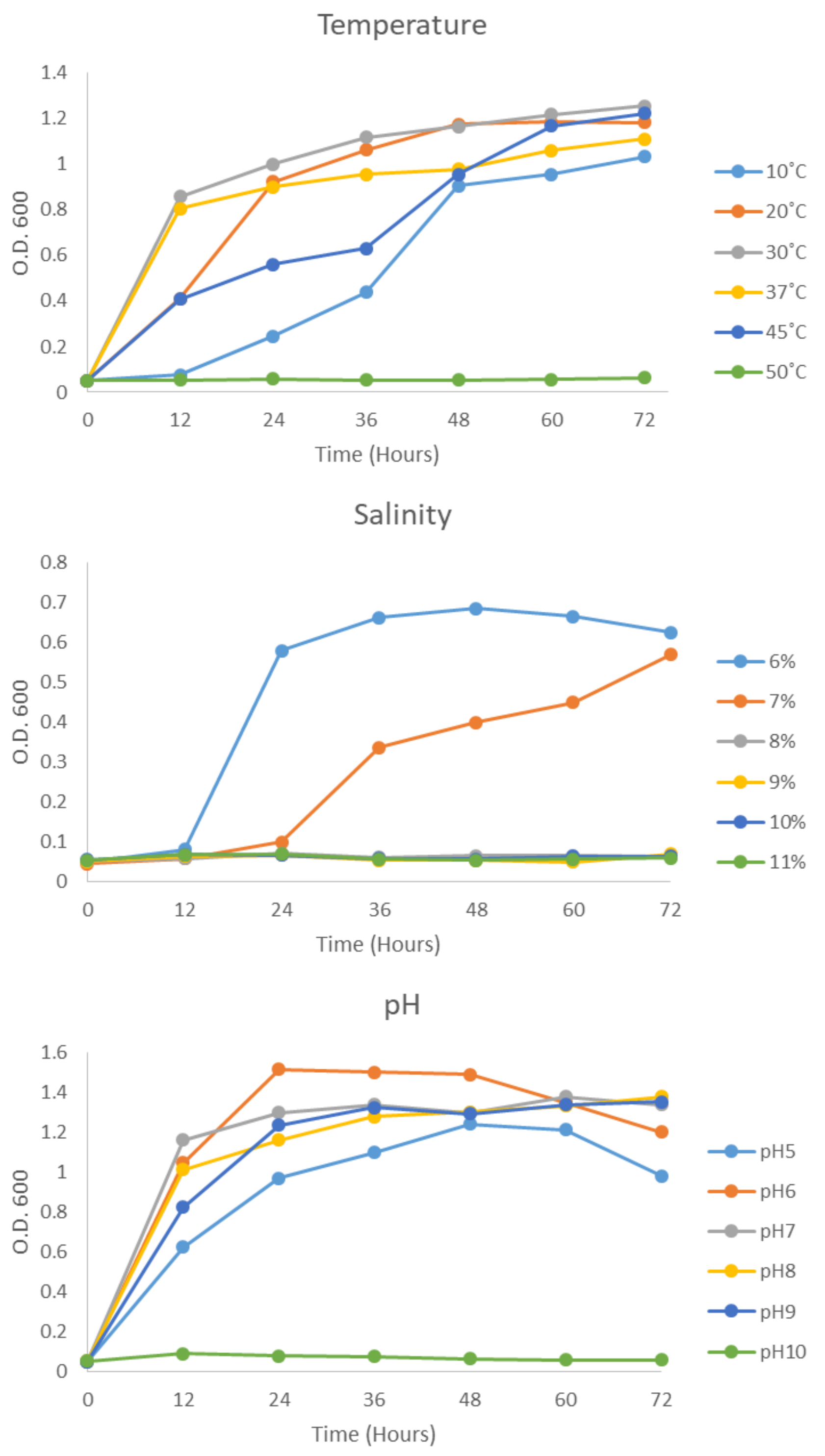


**Figure S3.** Growth optimization of *Enterobacter Sp*. LR6 at different temperature (**a**), salinity (**b**), and pH (**c**).

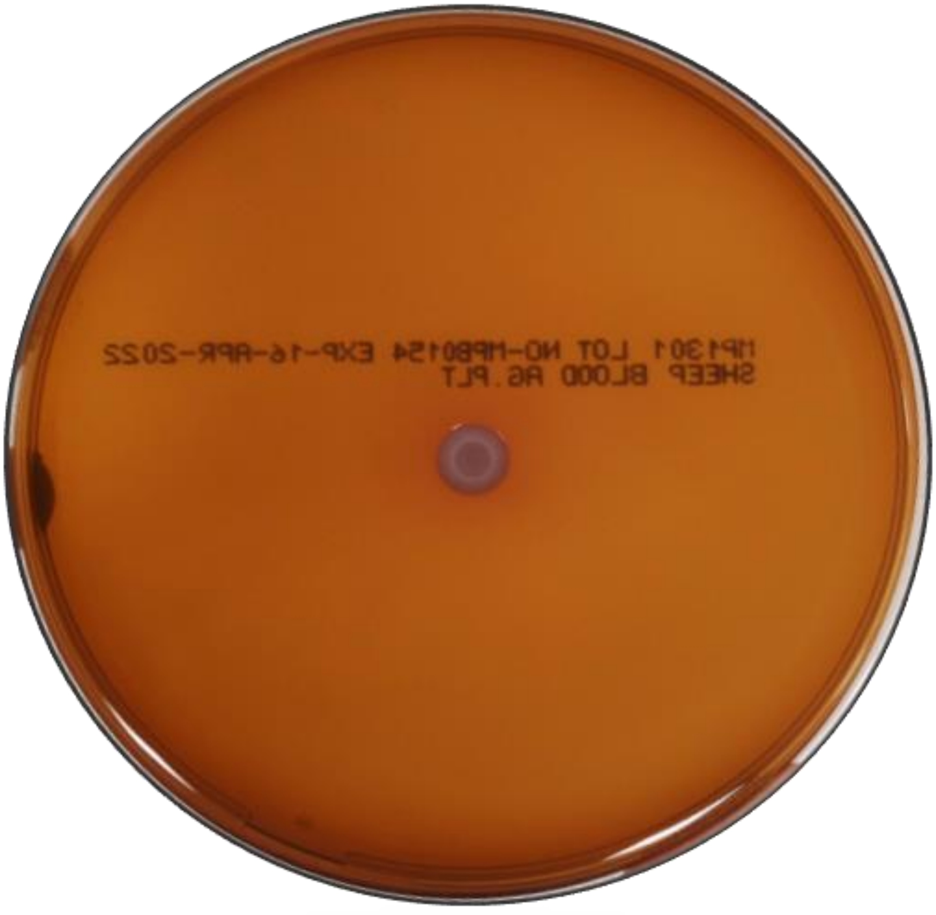


**Figure S4.** Pathogenicity testing using sheep blood agar plate, the absence of hemolytic zone around colony indicate LR6 is nonpathogenic

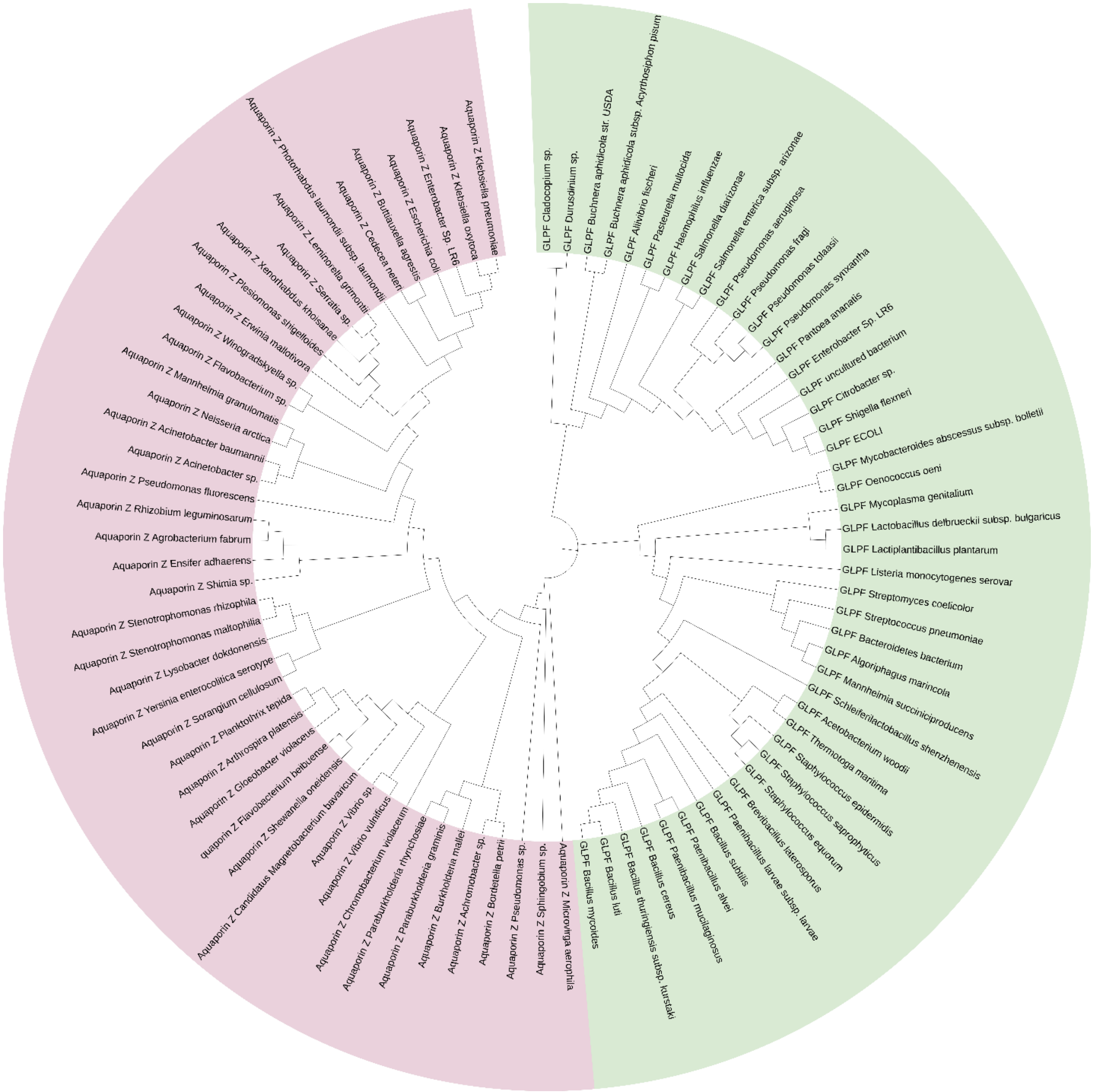


**Figure S5.** The phylogenetic tree constructed using maximum likelihood methods to elucidate the evolutionary relationship between GlpF and AQPZ sequences of different bacteria.
