# Supplementary material for: Genomic Landscape Highlights Molecular Mechanisms Involved in Silicate Solubilization, Stress Tolerance, and Potential Growth-Promoting Activity of Bacterium Enterobacter sp. LR6
Source: Cells. 2022 Nov 15;11(22):3622. doi: 10.3390/cells11223622 (PMC9688052; doi:10.3390/cells11223622)
Supplement: Supplementary file 1 [file cells-11-03622-s001.zip › cells-1917087-supplementary/cells-1917087-revised SM/Table S1.pdf]

Table S1: List of all the predicted transporters present in LR6 genome

ORF Name	Type	Transporter Family	Subfamily	Substrate/Function
sequence01_4440677_4442038	2-HCT	The 2-Hydroxycarboxylate Transporter (2-HCT) Family	-	sodium ion:citrate/malate symporter
sequence01_3016244_3017905	AAE	The Aspartate:Alanine Exchanger (AAE) Family	-	aspartate:alanine antiporter
sequence01_676044_674272	AAE	The Aspartate:Alanine Exchanger (AAE) Family	-	aspartate:alanine antiporter
sequence01_1000799_1000179	ABC	The ATP-binding Cassette (ABC) Superfamily	binding	sulfate
sequence01_1002328_1000799	ABC	The ATP-binding Cassette (ABC) Superfamily	membrane	-
sequence01_1003470_1002301	ABC	The ATP-binding Cassette (ABC) Superfamily	binding protein	spermidine/putrescine
sequence01_1040054_1041034	ABC	The ATP-binding Cassette (ABC) Superfamily	membrane	Vitamin B12
sequence01_1041619_1042374	ABC	The ATP-binding Cassette (ABC) Superfamily	binding	molybdate
sequence01_1046197_1045418	ABC	The ATP-binding Cassette (ABC) Superfamily	binding	phosphonate
sequence01_1047182_1046190	ABC	The ATP-binding Cassette (ABC) Superfamily	membrane	cobalamin/Fe3+-siderophores
sequence01_1048000_1047182	ABC	The ATP-binding Cassette (ABC) Superfamily	binding	iron-hydroxamate
sequence01_1080966_1082456	ABC	The ATP-binding Cassette (ABC) Superfamily	membrane	(Fe-S assembly/SufBCD system)
sequence01_1082466_1083212	ABC	The ATP-binding Cassette (ABC) Superfamily	ABC	(Fe-S assembly/SufBCD system)
sequence01_1083187_1084458	ABC	The ATP-binding Cassette (ABC) Superfamily	membrane	(Fe-S assembly/SufBCD system)
sequence01_1092553_1093575	ABC	The ATP-binding Cassette (ABC) Superfamily	binding	D-methionine
sequence01_1093568_1094236	ABC	The ATP-binding Cassette (ABC) Superfamily	membrane	D-methionine
sequence01_1094260_1095072	ABC	The ATP-binding Cassette (ABC) Superfamily	binding	methionine
sequence01_1096057_1095371	ABC	The ATP-binding Cassette (ABC) Superfamily	membrane	amino aicd (glutamine/glutamate/aspartate)
sequence01_1096787_1096041	ABC	The ATP-binding Cassette (ABC) Superfamily	membrane	amino aicd (glutamine/glutamate/aspartate)
sequence01_1097602_1096787	ABC	The ATP-binding Cassette (ABC) Superfamily	binding	phosphonate
sequence01_1098440_1097604	ABC	The ATP-binding Cassette (ABC) Superfamily	binding protein	amino acid (glutamine/glutamate/aspartate)
sequence01_1252716_1251568	ABC	The ATP-binding Cassette (ABC) Superfamily	binding	glycine betaine
sequence01_1253363_1252716	ABC	The ATP-binding Cassette (ABC) Superfamily	membrane	glycine betaine/L-proline/carnitine/choline
sequence01_1254271_1253372	ABC	The ATP-binding Cassette (ABC) Superfamily	binding	glycine betaine
sequence01_1255000_1254290	ABC	The ATP-binding Cassette (ABC) Superfamily	membrane	glycine betaine/L-proline/carnitine/choline

sequence01_1330353_1331267	ABC	The ATP-binding Cassette (ABC) Superfamily	binding	tungstate/molybdate
sequence01_1331282_1332028	ABC	The ATP-binding Cassette (ABC) Superfamily	membrane	molybdate
sequence01_135679_136641	ABC	The ATP-binding Cassette (ABC) Superfamily	binding	glycine betaine
sequence01_136652_137419	ABC	The ATP-binding Cassette (ABC) Superfamily	binding	nitrate
sequence01_137416_138243	ABC	The ATP-binding Cassette (ABC) Superfamily	membrane	nitrate/sulfonate/taurine
sequence01_1415883_1418048	ABC	The ATP-binding Cassette (ABC) Superfamily	ABC+ membrane	toxin secretion
sequence01_1434895_1435668	ABC	The ATP-binding Cassette (ABC) Superfamily	binding protein	amino acid (glutamine/glutamate/aspartate)
sequence01_1442212_1443807	ABC	The ATP-binding Cassette (ABC) Superfamily	binding protein	dipeptide/oligopeptide
sequence01_1454333_1455604	ABC	The ATP-binding Cassette (ABC) Superfamily	binding	urea
sequence01_1455627_1457201	ABC	The ATP-binding Cassette (ABC) Superfamily	membrane	branched-chain amino acid
sequence01_1457201_1458274	ABC	The ATP-binding Cassette (ABC) Superfamily	membrane	branched-chain amino acid
sequence01_1458274_1459071	ABC	The ATP-binding Cassette (ABC) Superfamily	binding	daunorubicin
sequence01_1459081_1459779	ABC	The ATP-binding Cassette (ABC) Superfamily	binding	molybdate
sequence01_1479659_1480303	ABC	The ATP-binding Cassette (ABC) Superfamily	membrane	-
sequence01_1481571_1483778	ABC	The ATP-binding Cassette (ABC) Superfamily	ABC+ membrane	toxin secretion
sequence01_1589426_1590958	ABC	The ATP-binding Cassette (ABC) Superfamily	binding protein	dipeptide/oligopeptide
sequence01_1590982_1592004	ABC	The ATP-binding Cassette (ABC) Superfamily	membrane	dipeptide/oligopeptide
sequence01_1592001_1592897	ABC	The ATP-binding Cassette (ABC) Superfamily	membrane	dipeptide/oligopeptide
sequence01_1592894_1593880	ABC	The ATP-binding Cassette (ABC) Superfamily	binding	glycine betaine
sequence01_1593873_1594799	ABC	The ATP-binding Cassette (ABC) Superfamily	binding	glycine betaine
sequence01_1602340_1604049	ABC	The ATP-binding Cassette (ABC) Superfamily	binding	lipoprotein
sequence01_1637593_1636820	ABC	The ATP-binding Cassette (ABC) Superfamily	binding	phosphate
sequence01_1638503_1637577	ABC	The ATP-binding Cassette (ABC) Superfamily	membrane	amino aicd (glutamine/glutamate/aspartate)
sequence01_1639964_1639032	ABC	The ATP-binding Cassette (ABC) Superfamily	binding protein	amino acid (glutamine/glutamate/aspartate)
sequence01_1656671_1655073	ABC	The ATP-binding Cassette (ABC) Superfamily	binding protein	dipeptide/oligopeptide

sequence01_1692798_1691182	ABC	The ATP-binding Cassette (ABC) Superfamily	binding protein	oligopeptide
sequence01_1702660_1701578	ABC	The ATP-binding Cassette (ABC) Superfamily	binding	polyamine
sequence01_1709400_1708558	ABC	The ATP-binding Cassette (ABC) Superfamily	membrane	sugar
sequence01_1710268_1709387	ABC	The ATP-binding Cassette (ABC) Superfamily	membrane	sugar
sequence01_1711582_1710290	ABC	The ATP-binding Cassette (ABC) Superfamily	binding protein	sugar
sequence01_1716260_1717900	ABC	The ATP-binding Cassette (ABC) Superfamily	binding protein	oligopeptide
sequence01_1717897_1718862	ABC	The ATP-binding Cassette (ABC) Superfamily	membrane	antimicrobial peptide uptake
sequence01_1718849_1719739	ABC	The ATP-binding Cassette (ABC) Superfamily	membrane	antimicrobial peptide uptake
sequence01_1719739_1720731	ABC	The ATP-binding Cassette (ABC) Superfamily	binding	phosphonate
sequence01_1720733_1721539	ABC	The ATP-binding Cassette (ABC) Superfamily	binding	glycine betaine
sequence01_1772790_1771786	ABC	The ATP-binding Cassette (ABC) Superfamily	binding	glycine betaine
sequence01_1773800_1772787	ABC	The ATP-binding Cassette (ABC) Superfamily	binding	lipoprotein
sequence01_1774720_1773812	ABC	The ATP-binding Cassette (ABC) Superfamily	membrane	dipeptide/oligopeptide
sequence01_1775653_1774733	ABC	The ATP-binding Cassette (ABC) Superfamily	membrane	dipeptide/oligopeptide
sequence01_1777367_1775736	ABC	The ATP-binding Cassette (ABC) Superfamily	binding protein	oligopeptide
sequence01_1809001_1808213	ABC	The ATP-binding Cassette (ABC) Superfamily	binding	nitrate
sequence01_1809889_1809011	ABC	The ATP-binding Cassette (ABC) Superfamily	membrane	nitrate/sulfonate/taurine
sequence01_1948467_1947508	ABC	The ATP-binding Cassette (ABC) Superfamily	binding	zinc
sequence01_1948530_1949285	ABC	The ATP-binding Cassette (ABC) Superfamily	binding	daunorubicin
sequence01_1949282_1950067	ABC	The ATP-binding Cassette (ABC) Superfamily	membrane	manganese/zinc ion
sequence01_1992918_1991938	ABC	The ATP-binding Cassette (ABC) Superfamily	membrane	sugar (ribose)
sequence01_1994447_1992933	ABC	The ATP-binding Cassette (ABC) Superfamily	ABC	sugar (ribose)
sequence01_1995508_1994519	ABC	The ATP-binding Cassette (ABC) Superfamily	binding	ribose
sequence01_2006500_2007621	ABC	The ATP-binding Cassette (ABC) Superfamily	binding	leucine/valine
sequence01_2007690_2008604	ABC	The ATP-binding Cassette (ABC) Superfamily	membrane	branched-chain amino acid
sequence01_2008616_2009890	ABC	The ATP-binding Cassette (ABC) Superfamily	membrane	branched-chain amino acid
sequence01_2009887_2010762	ABC	The ATP-binding Cassette (ABC) Superfamily	binding	daunorubicin

sequence01_2010771_2011475	ABC	The ATP-binding Cassette (ABC) Superfamily	binding	phosphate
sequence01_2019298_2018546	ABC	The ATP-binding Cassette (ABC) Superfamily	binding	phosphate
sequence01_2019963_2019295	ABC	The ATP-binding Cassette (ABC) Superfamily	membrane	amino aicd (glutamine/glutamate/aspartate)
sequence01_2021875_2021075	ABC	The ATP-binding Cassette (ABC) Superfamily	binding protein	amino acid (glutamine/glutamate/aspartate)
sequence01_209259_208309	ABC	The ATP-binding Cassette (ABC) Superfamily	binding	rhamnose
sequence01_2099746_2100534	ABC	The ATP-binding Cassette (ABC) Superfamily	binding	mobybdenate
sequence01_210289_209306	ABC	The ATP-binding Cassette (ABC) Superfamily	membrane	sugar (ribose)
sequence01_211791_210292	ABC	The ATP-binding Cassette (ABC) Superfamily	ABC	sugar (ribose)
sequence01_2217773_2217000	ABC	The ATP-binding Cassette (ABC) Superfamily	binding	D-methionine
sequence01_2218610_2217783	ABC	The ATP-binding Cassette (ABC) Superfamily	ABC	dipeptide/oligopeptide
sequence01_2219520_2218615	ABC	The ATP-binding Cassette (ABC) Superfamily	membrane	dipeptide/oligopeptide
sequence01_2220554_2219517	ABC	The ATP-binding Cassette (ABC) Superfamily	membrane	dipeptide/oligopeptide
sequence01_2222167_2220551	ABC	The ATP-binding Cassette (ABC) Superfamily	binding protein	dipeptide/oligopeptide
sequence01_2252431_2251694	ABC	The ATP-binding Cassette (ABC) Superfamily	membrane	glycine betaine/L-proline/carnitine/choline
sequence01_2253365_2252415	ABC	The ATP-binding Cassette (ABC) Superfamily	binding	glycine betaine
sequence01_2254515_2253358	ABC	The ATP-binding Cassette (ABC) Superfamily	membrane	glycine betaine/L-proline/carnitine/choline
sequence01_2255445_2254528	ABC	The ATP-binding Cassette (ABC) Superfamily	binding	glycine betaine
sequence01_2270832_2269822	ABC	The ATP-binding Cassette (ABC) Superfamily	membrane	D-galactose/galactoside
sequence01_2272368_2270848	ABC	The ATP-binding Cassette (ABC) Superfamily	ABC	sugar (ribose)
sequence01_2273446_2272448	ABC	The ATP-binding Cassette (ABC) Superfamily	binding	xylose
sequence01_2299797_2301626	ABC	The ATP-binding Cassette (ABC) Superfamily	binding protein	oligopeptide
sequence01_2301636_2302730	ABC	The ATP-binding Cassette (ABC) Superfamily	membrane	dipeptide/oligopeptide
sequence01_2302730_2303755	ABC	The ATP-binding Cassette (ABC) Superfamily	membrane	dipeptide/oligopeptide
sequence01_2303757_2305346	ABC	The ATP-binding Cassette (ABC) Superfamily	binding	glycine betaine
sequence01_2321055_2319412	ABC	The ATP-binding Cassette (ABC) Superfamily	ABC+ membrane	pyoverdin (siderophore) exporter PvdE
sequence01_239060_240814	ABC	The ATP-binding Cassette (ABC) Superfamily	binding	lipid A
sequence01_2404387_2403611	ABC	The ATP-binding Cassette (ABC) Superfamily	binding	phosphate

sequence01_2405107_2404394	ABC	The ATP-binding Cassette (ABC) Superfamily	membrane	amino acid (lysine/arginine/ornithine/histidine/octopine)
sequence01_2405790_2405104	ABC	The ATP-binding Cassette (ABC) Superfamily	membrane	amino acid (lysine/arginine/ornithine/histidine/octopine)
sequence01_2406661_2405879	ABC	The ATP-binding Cassette (ABC) Superfamily	binding protein	amino acid (glutamine/glutamate/aspartate)
sequence01_2407670_2406888	ABC	The ATP-binding Cassette (ABC) Superfamily	binding protein	amino acid (glutamine/glutamate/aspartate)
sequence01_240807_242588	ABC	The ATP-binding Cassette (ABC) Superfamily	binding	lipid A
sequence01_2472520_2471426	ABC	The ATP-binding Cassette (ABC) Superfamily	binding	sulfate
sequence01_2473385_2472510	ABC	The ATP-binding Cassette (ABC) Superfamily	membrane	sulfate
sequence01_2474218_2473385	ABC	The ATP-binding Cassette (ABC) Superfamily	membrane	sulfate
sequence01_2475231_2474218	ABC	The ATP-binding Cassette (ABC) Superfamily	binding	sulfate/thiosulfate
sequence01_2549198_2547732	ABC	The ATP-binding Cassette (ABC) Superfamily	binding	glycine betaine
sequence01_2607449_2608999	ABC	The ATP-binding Cassette (ABC) Superfamily	binding protein	amino acid
sequence01_2689305_2691209	ABC	The ATP-binding Cassette (ABC) Superfamily	binding	cobalt
sequence01_2782933_2781863	ABC	The ATP-binding Cassette (ABC) Superfamily	binding	polyamine
sequence01_2783780_2782935	ABC	The ATP-binding Cassette (ABC) Superfamily	membrane	sugar
sequence01_2784664_2783777	ABC	The ATP-binding Cassette (ABC) Superfamily	membrane	sugar
sequence01_2786138_2784822	ABC	The ATP-binding Cassette (ABC) Superfamily	binding protein	sugar
sequence01_2787099_2786386	ABC	The ATP-binding Cassette (ABC) Superfamily	binding	daunorubicin
sequence01_2787865_2787101	ABC	The ATP-binding Cassette (ABC) Superfamily	binding	daunorubicin
sequence01_2789139_2787862	ABC	The ATP-binding Cassette (ABC) Superfamily	membrane	branched-chain amino acid
sequence01_2790062_2789136	ABC	The ATP-binding Cassette (ABC) Superfamily	membrane	branched-chain amino acid
sequence01_2791218_2790109	ABC	The ATP-binding Cassette (ABC) Superfamily	binding	leucine/valine
sequence01_2793283_2792189	ABC	The ATP-binding Cassette (ABC) Superfamily	binding	leucine/valine
sequence01_2797052_2795997	ABC	The ATP-binding Cassette (ABC) Superfamily	membrane	cell division
sequence01_2797710_2797045	ABC	The ATP-binding Cassette (ABC) Superfamily	binding	lipoprotein
sequence01_2895515_2896501	ABC	The ATP-binding Cassette (ABC) Superfamily	binding	rhamnose
sequence01_2896601_2898103	ABC	The ATP-binding Cassette (ABC) Superfamily	ABC	sugar (ribose)

sequence01_2898103_2899107	ABC	The ATP-binding Cassette (ABC) Superfamily	membrane	sugar (ribose)
sequence01_2899116_2900108	ABC	The ATP-binding Cassette (ABC) Superfamily	membrane	sugar (ribose)
sequence01_293118_293789	ABC	The ATP-binding Cassette (ABC) Superfamily	binding	lipoprotein
sequence01_293782_294564	ABC	The ATP-binding Cassette (ABC) Superfamily	membrane	-
sequence01_2958806_2957916	ABC	The ATP-binding Cassette (ABC) Superfamily	binding	ribose
sequence01_2959799_2958834	ABC	The ATP-binding Cassette (ABC) Superfamily	membrane	sugar (ribose)
sequence01_2961309_2959804	ABC	The ATP-binding Cassette (ABC) Superfamily	ABC	sugar (ribose)
sequence01_296922_297608	ABC	The ATP-binding Cassette (ABC) Superfamily	binding	lipoprotein
sequence01_297605_300019	ABC	The ATP-binding Cassette (ABC) Superfamily	membrane	-
sequence01_2983463_2984503	ABC	The ATP-binding Cassette (ABC) Superfamily	binding protein	phosphate
sequence01_2984631_2985590	ABC	The ATP-binding Cassette (ABC) Superfamily	membrane	phosphate
sequence01_2985590_2986480	ABC	The ATP-binding Cassette (ABC) Superfamily	membrane	phosphate
sequence01_2986528_2987301	ABC	The ATP-binding Cassette (ABC) Superfamily	binding	phosphate
sequence01_3072909_3073709	ABC	The ATP-binding Cassette (ABC) Superfamily	binding	methionine
sequence01_3089880_3091976	ABC	The ATP-binding Cassette (ABC) Superfamily	ABC+ membrane	toxin secretion
sequence01_3236820_3238439	ABC	The ATP-binding Cassette (ABC) Superfamily	binding	lipoprotein
sequence01_3254701_3256308	ABC	The ATP-binding Cassette (ABC) Superfamily	binding protein	dipeptide/oligopeptide
sequence01_3256470_3257489	ABC	The ATP-binding Cassette (ABC) Superfamily	membrane	dipeptide/oligopeptide
sequence01_3257499_3258401	ABC	The ATP-binding Cassette (ABC) Superfamily	membrane	dipeptide/oligopeptide
sequence01_3258412_3259395	ABC	The ATP-binding Cassette (ABC) Superfamily	binding	glycine betaine
sequence01_3259392_3260405	ABC	The ATP-binding Cassette (ABC) Superfamily	binding	glycine betaine
sequence01_3392454_3393410	ABC	The ATP-binding Cassette (ABC) Superfamily	binding	ribose
sequence01_3393514_3395016	ABC	The ATP-binding Cassette (ABC) Superfamily	ABC	sugar (ribose)
sequence01_3395030_3396052	ABC	The ATP-binding Cassette (ABC) Superfamily	membrane	sugar (ribose)
sequence01_3396039_3397040	ABC	The ATP-binding Cassette (ABC) Superfamily	membrane	sugar (ribose)
sequence01_3542542_3540875	ABC	The ATP-binding Cassette (ABC) Superfamily	binding	sulfate
sequence01_3614427_3613729	ABC	The ATP-binding Cassette (ABC) Superfamily	binding	thiamine
sequence01_3616021_3614411	ABC	The ATP-binding Cassette (ABC) Superfamily	membrane	iron(III)

sequence01_3616980_3615997	ABC	The ATP-binding Cassette (ABC) Superfamily	binding	thiamin
sequence01_363572_362868	ABC	The ATP-binding Cassette (ABC) Superfamily	binding	polyamine
sequence01_364395_363559	ABC	The ATP-binding Cassette (ABC) Superfamily	binding	lipoprotein
sequence01_365221_364388	ABC	The ATP-binding Cassette (ABC) Superfamily	membrane	dipeptide/oligopeptide
sequence01_366234_365221	ABC	The ATP-binding Cassette (ABC) Superfamily	membrane	dipeptide/oligopeptide
sequence01_367901_366333	ABC	The ATP-binding Cassette (ABC) Superfamily	binding protein	dipeptide/oligopeptide
sequence01_3687292_3688218	ABC	The ATP-binding Cassette (ABC) Superfamily	binding	daunorubicin
sequence01_3688215_3688985	ABC	The ATP-binding Cassette (ABC) Superfamily	membrane	polysaccharide export
sequence01_3717845_3718642	ABC	The ATP-binding Cassette (ABC) Superfamily	binding	phosphonate
sequence01_3718642_3719532	ABC	The ATP-binding Cassette (ABC) Superfamily	binding	iron-hydroxamate
sequence01_3719529_3721511	ABC	The ATP-binding Cassette (ABC) Superfamily	membrane	cobalamin/Fe3+-siderophores
sequence01_3726426_3725617	ABC	The ATP-binding Cassette (ABC) Superfamily	binding	iron-hydroxamate
sequence01_3766639_3765824	ABC	The ATP-binding Cassette (ABC) Superfamily	binding	methionine
sequence01_3767333_3766680	ABC	The ATP-binding Cassette (ABC) Superfamily	membrane	D-methionine
sequence01_3768357_3767326	ABC	The ATP-binding Cassette (ABC) Superfamily	binding	D-methionine
sequence01_377351_378262	ABC	The ATP-binding Cassette (ABC) Superfamily	binding	zinc
sequence01_3781836_3780841	ABC	The ATP-binding Cassette (ABC) Superfamily	binding	glycine betaine
sequence01_378265_379071	ABC	The ATP-binding Cassette (ABC) Superfamily	binding	phosphonate
sequence01_3782910_3781846	ABC	The ATP-binding Cassette (ABC) Superfamily	membrane	glycine betaine/L-proline
sequence01_3784105_3782903	ABC	The ATP-binding Cassette (ABC) Superfamily	binding	glycine betaine
sequence01_379068_379919	ABC	The ATP-binding Cassette (ABC) Superfamily	membrane	manganese/zinc ion
sequence01_379913_380752	ABC	The ATP-binding Cassette (ABC) Superfamily	membrane	manganese/zinc ion
sequence01_3857071_3858060	ABC	The ATP-binding Cassette (ABC) Superfamily	binding	sulfate/thiosulfate
sequence01_390002_389202	ABC	The ATP-binding Cassette (ABC) Superfamily	binding	phosphonate
sequence01_390991_389999	ABC	The ATP-binding Cassette (ABC) Superfamily	membrane	ferric enterobactin
sequence01_391992_390988	ABC	The ATP-binding Cassette (ABC) Superfamily	membrane	cobalamin/Fe3+-siderophores
sequence01_3926413_3925523	ABC	The ATP-binding Cassette (ABC) Superfamily	membrane	sugar (maltose)
sequence01_3927972_3926428	ABC	The ATP-binding Cassette (ABC) Superfamily	membrane	sugar

sequence01_3929284_3928094	ABC	The ATP-binding Cassette (ABC) Superfamily	binding protein	sugar (maltose)
sequence01_3929656_3930765	ABC	The ATP-binding Cassette (ABC) Superfamily	binding	polyamine
sequence01_394365_393406	ABC	The ATP-binding Cassette (ABC) Superfamily	binding	iron-hydroxamate
sequence01_3981708_3980767	ABC	The ATP-binding Cassette (ABC) Superfamily	binding	xylose
sequence01_3982732_3981737	ABC	The ATP-binding Cassette (ABC) Superfamily	membrane	ribose
sequence01_3984249_3982735	ABC	The ATP-binding Cassette (ABC) Superfamily	ABC	sugar (ribose)
sequence01_3989275_3988295	ABC	The ATP-binding Cassette (ABC) Superfamily	membrane	sugar (ribose)
sequence01_3990786_3989254	ABC	The ATP-binding Cassette (ABC) Superfamily	ABC	sugar (ribose)
sequence01_3991798_3990863	ABC	The ATP-binding Cassette (ABC) Superfamily	binding	rhamnose
sequence01_3997558_3996878	ABC	The ATP-binding Cassette (ABC) Superfamily	ABC	phosphonate
sequence01_3998471_3997716	ABC	The ATP-binding Cassette (ABC) Superfamily	ABC	phosphonate
sequence01_4002929_4002150	ABC	The ATP-binding Cassette (ABC) Superfamily	membrane	phosphonate
sequence01_4004093_4003077	ABC	The ATP-binding Cassette (ABC) Superfamily	binding	phosphonate
sequence01_4004905_4004117	ABC	The ATP-binding Cassette (ABC) Superfamily	binding	phosphonate
sequence01_404802_406304	ABC	The ATP-binding Cassette (ABC) Superfamily	ABC	sugar (ribose)
sequence01_406301_407296	ABC	The ATP-binding Cassette (ABC) Superfamily	membrane	sugar (ribose)
sequence01_407316_408380	ABC	The ATP-binding Cassette (ABC) Superfamily	binding	rhamnose
sequence01_4115907_4115182	ABC	The ATP-binding Cassette (ABC) Superfamily	binding	daunorubicin
sequence01_4119795_4120607	ABC	The ATP-binding Cassette (ABC) Superfamily	binding	glycine betaine
sequence01_4120615_4121397	ABC	The ATP-binding Cassette (ABC) Superfamily	membrane	toluene tolerance
sequence01_4121402_4121950	ABC	The ATP-binding Cassette (ABC) Superfamily	binding protein	toluene tolerance
sequence01_4121969_4122604	ABC	The ATP-binding Cassette (ABC) Superfamily	binding	toluene tolerance
sequence01_4220323_4221810	ABC	The ATP-binding Cassette (ABC) Superfamily	ABC	sugar (ribose)
sequence01_4221807_4222838	ABC	The ATP-binding Cassette (ABC) Superfamily	membrane	sugar (ribose)
sequence01_4222839_4223816	ABC	The ATP-binding Cassette (ABC) Superfamily	membrane	sugar (ribose)
sequence01_4223818_4224819	ABC	The ATP-binding Cassette (ABC) Superfamily	binding	rhamnose
sequence01_4268592_4271042	ABC	The ATP-binding Cassette (ABC) Superfamily	binding	iron-hydroxamate
sequence01_4315510_4316322	ABC	The ATP-binding Cassette (ABC) Superfamily	binding	phosphonate

sequence01_4316319_4317302	ABC	The ATP-binding Cassette (ABC) Superfamily	membrane	cobalamin/Fe3+-siderophores
sequence01_4317299_4318339	ABC	The ATP-binding Cassette (ABC) Superfamily	membrane	cobalamin/Fe3+-siderophores
sequence01_4564728_4563442	ABC	The ATP-binding Cassette (ABC) Superfamily	binding protein	sugar
sequence01_4565869_4564742	ABC	The ATP-binding Cassette (ABC) Superfamily	binding	polyamine
sequence01_4566784_4565882	ABC	The ATP-binding Cassette (ABC) Superfamily	membrane	sugar
sequence01_4567667_4566777	ABC	The ATP-binding Cassette (ABC) Superfamily	membrane	sugar
sequence01_461489_460764	ABC	The ATP-binding Cassette (ABC) Superfamily	binding	phosphate
sequence01_462163_461489	ABC	The ATP-binding Cassette (ABC) Superfamily	membrane	amino aicd (glutamine/glutamate/aspartate)
sequence01_462904_462164	ABC	The ATP-binding Cassette (ABC) Superfamily	membrane	amino aicd (glutamine/glutamate/aspartate)
sequence01_463965_463060	ABC	The ATP-binding Cassette (ABC) Superfamily	binding protein	amino acid (glutamine/glutamate/aspartate)
sequence01_4708739_4709392	ABC	The ATP-binding Cassette (ABC) Superfamily	binding	phosphonate
sequence01_4709389_4710249	ABC	The ATP-binding Cassette (ABC) Superfamily	membrane	manganese/zinc ion
sequence01_4710285_4711142	ABC	The ATP-binding Cassette (ABC) Superfamily	binding	zinc
sequence01_483477_482410	ABC	The ATP-binding Cassette (ABC) Superfamily	binding protein	sugar
sequence01_551960_550488	ABC	The ATP-binding Cassette (ABC) Superfamily	binding	daunorubicin
sequence01_553275_554051	ABC	The ATP-binding Cassette (ABC) Superfamily	binding	mobybdenate
sequence01_554048_554740	ABC	The ATP-binding Cassette (ABC) Superfamily	membrane	molybdate
sequence01_554740_555798	ABC	The ATP-binding Cassette (ABC) Superfamily	binding	molybdate
sequence01_56734_55553	ABC	The ATP-binding Cassette (ABC) Superfamily	membrane	sugar (xylose)
sequence01_571885_571163	ABC	The ATP-binding Cassette (ABC) Superfamily	binding	molybdate
sequence01_58253_56712	ABC	The ATP-binding Cassette (ABC) Superfamily	ABC	sugar (ribose)
sequence01_584154_583048	ABC	The ATP-binding Cassette (ABC) Superfamily	membrane	multidrug
sequence01_585345_584212	ABC	The ATP-binding Cassette (ABC) Superfamily	membrane	multidrug
sequence01_587074_585335	ABC	The ATP-binding Cassette (ABC) Superfamily	binding	daunorubicin
sequence01_59318_58326	ABC	The ATP-binding Cassette (ABC) Superfamily	binding	xylose
sequence01_604741_604019	ABC	The ATP-binding Cassette (ABC) Superfamily	binding	phosphate
sequence01_605397_604738	ABC	The ATP-binding Cassette (ABC) Superfamily	membrane	amino aicd (glutamine/glutamate/aspartate)

sequence01_606234_605491	ABC	The ATP-binding Cassette (ABC) Superfamily	binding protein	amino acid (glutamine/glutamate/aspartate)
sequence01_627031_623372	ABC	The ATP-binding Cassette (ABC) Superfamily	binding	lipid A
sequence01_630140_632080	ABC	The ATP-binding Cassette (ABC) Superfamily	binding	lipoprotein
sequence01_638305_639900	ABC	The ATP-binding Cassette (ABC) Superfamily	binding	heme
sequence01_654924_656795	ABC	The ATP-binding Cassette (ABC) Superfamily	binding	glycine betaine
sequence01_656819_658357	ABC	The ATP-binding Cassette (ABC) Superfamily	binding protein	dipeptide/oligopeptide
sequence01_658363_659283	ABC	The ATP-binding Cassette (ABC) Superfamily	membrane	dipeptide/oligopeptide
sequence01_659285_660196	ABC	The ATP-binding Cassette (ABC) Superfamily	membrane	dipeptide/oligopeptide
sequence01_679672_680784	ABC	The ATP-binding Cassette (ABC) Superfamily	binding protein	spermidine/putrescine
sequence01_680928_682061	ABC	The ATP-binding Cassette (ABC) Superfamily	binding	polyamine
sequence01_682071_683024	ABC	The ATP-binding Cassette (ABC) Superfamily	membrane	spermidine/putrescine
sequence01_683021_683866	ABC	The ATP-binding Cassette (ABC) Superfamily	membrane	spermidine/putrescine
sequence01_688859_688128	ABC	The ATP-binding Cassette (ABC) Superfamily	binding protein	amino acid (glutamine/glutamate/aspartate)
sequence01_689707_689039	ABC	The ATP-binding Cassette (ABC) Superfamily	membrane	amino acid (lysine/arginine/ornithine/histidine/octopine)
sequence01_690423_689707	ABC	The ATP-binding Cassette (ABC) Superfamily	membrane	amino acid (lysine/arginine/ornithine/histidine/octopine)
sequence01_691161_690430	ABC	The ATP-binding Cassette (ABC) Superfamily	binding protein	amino acid (glutamine/glutamate/aspartate)
sequence01_691908_691180	ABC	The ATP-binding Cassette (ABC) Superfamily	binding	phosphate
sequence01_727942_729882	ABC	The ATP-binding Cassette (ABC) Superfamily	binding	lipoprotein
sequence01_736248_734527	ABC	The ATP-binding Cassette (ABC) Superfamily	binding	lipid A
sequence01_738014_736248	ABC	The ATP-binding Cassette (ABC) Superfamily	binding	lipid A
sequence01_769602_771350	ABC	The ATP-binding Cassette (ABC) Superfamily	binding	lipid A
sequence01_796720_795950	ABC	The ATP-binding Cassette (ABC) Superfamily	binding	nitrate
sequence01_797508_796717	ABC	The ATP-binding Cassette (ABC) Superfamily	membrane	nitrate/sulfonate/taurine
sequence01_799622_798660	ABC	The ATP-binding Cassette (ABC) Superfamily	binding protein	nitrate/sulfonate/taurine
sequence01_805485_807392	ABC	The ATP-binding Cassette (ABC) Superfamily	binding	heme

sequence01_827098_828726	ABC	The ATP-binding Cassette (ABC) Superfamily	binding protein	oligopeptide
sequence01_829780_829019	ABC	The ATP-binding Cassette (ABC) Superfamily	binding	sulfate
sequence01_830808_829777	ABC	The ATP-binding Cassette (ABC) Superfamily	membrane	cobalamin/Fe3+-siderophores
sequence01_831812_830805	ABC	The ATP-binding Cassette (ABC) Superfamily	binding	cobalamin
sequence01_85887_86900	ABC	The ATP-binding Cassette (ABC) Superfamily	binding	lipoprotein
sequence01_86897_87979	ABC	The ATP-binding Cassette (ABC) Superfamily	binding	glycine betaine
sequence01_87982_89496	ABC	The ATP-binding Cassette (ABC) Superfamily	binding protein	dipeptide/oligopeptide
sequence01_89515_90456	ABC	The ATP-binding Cassette (ABC) Superfamily	membrane	dipeptide/oligopeptide
sequence01_90459_91334	ABC	The ATP-binding Cassette (ABC) Superfamily	membrane	dipeptide/oligopeptide
sequence01_944744_945943	ABC	The ATP-binding Cassette (ABC) Superfamily	membrane	lipoprotein releasing
sequence01_945936_946637	ABC	The ATP-binding Cassette (ABC) Superfamily	binding	lipoprotein
sequence01_946637_947881	ABC	The ATP-binding Cassette (ABC) Superfamily	membrane	lipoprotein releasing
sequence01_950752_949718	ABC	The ATP-binding Cassette (ABC) Superfamily	binding protein	spermidine/putrescine
sequence01_951540_950749	ABC	The ATP-binding Cassette (ABC) Superfamily	membrane	spermidine/putrescine
sequence01_952394_951537	ABC	The ATP-binding Cassette (ABC) Superfamily	membrane	spermidine/putrescine
sequence01_953463_952378	ABC	The ATP-binding Cassette (ABC) Superfamily	binding	polyamine
sequence01_1216828_1215302	AbgT	The p-Aminobenzoyl-glutamate Transporter (AbgT) Family	-	aminobenzoyl-glutamate
sequence01_2694718_2693759	AEC	The Auxin Efflux Carrier (AEC) Family	-	
sequence01_3557854_3556421	AGCS	The Alanine or Glycine:Cation Symporter (AGCS) Family	-	sodium ion:alanine symporter
sequence01_862308_863813	AGCS	The Alanine or Glycine:Cation Symporter (AGCS) Family	-	sodium ion:alanine symporter
sequence01_1061714_1060599	AI-2E	The Autoinducer-2 Exporter (AI-2E) Family (Formerly the PerM Family, TC #9.B.22)	-	Autoinducer-2 export
sequence01_1241550_1240510	AI-2E	The Autoinducer-2 Exporter (AI-2E) Family (Formerly the PerM Family, TC #9.B.22)	-	Autoinducer-2 export
sequence01_132979_131870	AI-2E	The Autoinducer-2 Exporter (AI-2E) Family (Formerly the PerM Family, TC #9.B.22)	-	Autoinducer-2 export
sequence01_2515799_2514735	AI-2E	The Autoinducer-2 Exporter (AI-2E) Family (Formerly the PerM Family, TC #9.B.22)	-	Autoinducer-2 export

sequence01_2808579_2809628	AI-2E	The Autoinducer-2 Exporter (AI-2E) Family (Formerly the PerM Family, TC #9.B.22)	-	Autoinducer-2 export
sequence01_243174_244460	Amt	The Ammonia Transporter Channel (Amt) Family	-	ammonium
sequence01_1206901_1205519	APC	The Amino Acid-Polyamine-Organocation (APC) Family	-	arginine:ornithine antiporter
sequence01_133137_134537	APC	The Amino Acid-Polyamine-Organocation (APC) Family	-	amino acid
sequence01_1645881_1647377	APC	The Amino Acid-Polyamine-Organocation (APC) Family	-	amino acid
sequence01_170058_171428	APC	The Amino Acid-Polyamine-Organocation (APC) Family	-	amino acid
sequence01_2145246_2143888	APC	The Amino Acid-Polyamine-Organocation (APC) Family	-	amino acid
sequence01_2282757_2281288	APC	The Amino Acid-Polyamine-Organocation (APC) Family	-	amino acid
sequence01_2490768_2492204	APC	The Amino Acid-Polyamine-Organocation (APC) Family	-	glutamate:GABA antiporter
sequence01_3195718_3197103	APC	The Amino Acid-Polyamine-Organocation (APC) Family	-	GABA
sequence01_326434_327822	APC	The Amino Acid-Polyamine-Organocation (APC) Family	-	amino acid
sequence01_3304274_3303027	APC	The Amino Acid-Polyamine-Organocation (APC) Family	-	arginine:ornithine antiporter
sequence01_3320354_3321853	APC	The Amino Acid-Polyamine-Organocation (APC) Family	-	glutamate:GABA antiporter
sequence01_3364367_3365776	APC	The Amino Acid-Polyamine-Organocation (APC) Family	-	amino acid
sequence01_3665857_3664487	APC	The Amino Acid-Polyamine-Organocation (APC) Family	-	amino acid
sequence01_495767_494454	APC	The Amino Acid-Polyamine-Organocation (APC) Family	-	arginine:ornithine antiporter
sequence01_1125814_1123778	ArAE	The Aromatic Acid Exporter (ArAE) Family	-	fusaric acid efflux
sequence01_2140282_2139224	ArAE	The Aromatic Acid Exporter (ArAE) Family	-	fusaric acid efflux
sequence01_2703155_2705242	ArAE	The Aromatic Acid Exporter (ArAE) Family	-	fusaric acid efflux
sequence01_4066432_4068399	ArAE	The Aromatic Acid Exporter (ArAE) Family	-	fusaric acid efflux
sequence01_819294_817132	ArAE	The Aromatic Acid Exporter (ArAE) Family	-	fusaric acid efflux
sequence01_4734229_4735986	**ArsAB**	The Arsenite-Antimonite (ArsAB) Efflux Family	-	arsenite (ArsA)

sequence01_4742670_4740919	**ArsAB**	The Arsenite-Antimonite (ArsAB) Efflux Family	-	arsenite (ArsA)
sequence01_4740871_4739582	**ArsB**	The Arsenite-Antimonite (ArsB) Efflux Family	-	arsenite (ArsB)
sequence01_611908_613017	**ArsB**	The Arsenite-Antimonite (ArsB) Efflux Family	-	arsenite (ArsB)
sequence01_673660_672371	**ArsB**	The Arsenite-Antimonite (ArsB) Efflux Family	-	arsenite (ArsB)
sequence31_15724_17007	**ArsB**	The Arsenite-Antimonite (ArsB) Efflux Family	-	arsenite (ArsB)
sequence01_3917228_3916284	BASS	The Bile Acid:Na+ Symporter (BASS) Family	-	sodium ion/
sequence01_372774_374807	BCCT	The Betaine/Carnitine/Choline Transporter (BCCT) Family	-	glycine betaine/carnitine/choline
sequence01_1447740_1446574	BenE	The Benzoate:H+ Symporter (BenE) Family	-	benzoate
sequence01_1476934_1477848	Bestrophin	The Anion Channel-forming Bestrophin (Bestrophin) Family	-	Bestrophin anion channel
sequence01_1816694_1817794	CaCA	The Ca2+:Cation Antiporter (CaCA) Family	-	proton:calcium ion antiporter
sequence01_4119567_4118590	CaCA	The Ca2+:Cation Antiporter (CaCA) Family	-	sodium ion:calcium ion antiporter
sequence01_2884103_2883219	CDF	The Cation Diffusion Facilitator (CDF) Family	-	cation efflux
sequence01_543130_542192	CDF	The Cation Diffusion Facilitator (CDF) Family	-	cation efflux
sequence01_4722045_4720666	CHR	The Chromate Ion Transporter (CHR) Family	-	chromate ion
sequence01_2445708_2446943	ClC	The Chloride Carrier/Channel (ClC) Family	-	chloride ion channel
sequence01_3723081_3724481	ClC	The Chloride Carrier/Channel (ClC) Family	-	chloride ion channel
sequence01_2451879_2453066	CNT	The Concentrative Nucleoside Transporter (CNT) Family	-	sodium ion:nucleoside symporter
sequence01_857057_855873	CNT	The Concentrative Nucleoside Transporter (CNT) Family	-	sodium ion:nucleoside symporter
sequence01_1879396_1881129	CPA1	The Monovalent Cation:Proton Antiporter-1 (CPA1) Family	-	sodium ion:proton antiporter
sequence01_3962556_3964202	CPA1	The Monovalent Cation:Proton Antiporter-1 (CPA1) Family	-	sodium ion:proton antiporter
sequence01_2688621_2686816	CPA2	The Monovalent Cation:Proton Antiporter-2 (CPA2) Family	-	potassium/sodium ion:proton antiporter
sequence01_282706_281030	CPA2	The Monovalent Cation:Proton Antiporter-2 (CPA2) Family	-	potassium/sodium ion:proton antiporter
sequence01_3591093_3592958	CPA2	The Monovalent Cation:Proton Antiporter-2 (CPA2) Family	-	potassium/sodium ion:proton antiporter
sequence01_1025089_1023698	DAACS	The Dicarboxylate/Amino Acid:Cation (Na+ or H+) Symporter (DAACS) Family	-	proton/sodium ion:glutamate/aspartate symporter

sequence01_1981580_1982839	DAACS	The Dicarboxylate/Amino Acid:Cation (Na+ or H+) Symporter (DAACS) Family	-	proton/sodium ion:glutamate/aspartate symporter
sequence01_2862999_2861713	DAACS	The Dicarboxylate/Amino Acid:Cation (Na+ or H+) Symporter (DAACS) Family	-	proton/sodium ion:glutamate/aspartate symporter
sequence01_3970865_3972178	DAACS	The Dicarboxylate/Amino Acid:Cation (Na+ or H+) Symporter (DAACS) Family	-	proton/sodium ion:glutamate/aspartate symporter
sequence01_4210900_4209659	DAACS	The Dicarboxylate/Amino Acid:Cation (Na+ or H+) Symporter (DAACS) Family	-	sodium ion:serine/threonine symporter
sequence01_2092751_2094187	DASS	The Divalent Anion:Na+ Symporter (DASS) Family	-	sodium ion:dicarboxylate/sulfate
sequence01_2385419_2383587	DASS	The Divalent Anion:Na+ Symporter (DASS) Family	-	sodium ion:dicarboxylate/sulfate
sequence01_3858989_3860293	DASS	The Divalent Anion:Na+ Symporter (DASS) Family	-	sodium ion:dicarboxylate/sulfate
sequence01_1997966_1999309	Dcu	The C4-Dicarboxylate Uptake (Dcu) Family	-	C4-dicarboxylate
sequence01_3300650_3299349	Dcu	The C4-Dicarboxylate Uptake (Dcu) Family	-	C4-dicarboxylate
sequence01_3438169_3436766	DcuC	The C4-dicarboxylate Uptake C (DcuC) Family	-	C4-dicarboxylate
sequence01_434320_432953	DcuC	The C4-dicarboxylate Uptake C (DcuC) Family	-	C4-dicarboxylate
sequence01_1241972_1242334	DMT	The Drug/Metabolite Transporter (DMT) Superfamily	SMR	multidrug efflux (SMR)
sequence01_1242321_1242650	DMT	The Drug/Metabolite Transporter (DMT) Superfamily	SMR	multidrug efflux (SMR)
sequence01_1269690_1270016	DMT	The Drug/Metabolite Transporter (DMT) Superfamily	-	drug/metabolite
sequence01_1445547_1444651	DMT	The Drug/Metabolite Transporter (DMT) Superfamily	-	drug/metabolite
sequence01_1461928_1462827	DMT	The Drug/Metabolite Transporter (DMT) Superfamily	DME	drug/metabolite
sequence01_1614811_1615689	DMT	The Drug/Metabolite Transporter (DMT) Superfamily	-	drug/metabolite
sequence01_1654285_1654734	DMT	The Drug/Metabolite Transporter (DMT) Superfamily	-	drug/metabolite
sequence01_2079899_2080813	DMT	The Drug/Metabolite Transporter (DMT) Superfamily	DME	drug/metabolite
sequence01_2824154_2823771	DMT	The Drug/Metabolite Transporter (DMT) Superfamily	SMR	multidrug/quaternary ammonium compound efflux (SMR subfamily)
sequence01_2824474_2824151	DMT	The Drug/Metabolite Transporter (DMT) Superfamily	SMR	multidrug efflux (SMR)
sequence01_2888685_2889719	DMT	The Drug/Metabolite Transporter (DMT) Superfamily	-	drug/metabolite

sequence01_3039992_3039159	DMT	The Drug/Metabolite Transporter (DMT) Superfamily	GRP	glucose uptake (GRP subfamily)
sequence01_3055657_3054752	DMT	The Drug/Metabolite Transporter (DMT) Superfamily	DME	drug/metabolite
sequence01_3213780_3212884	DMT	The Drug/Metabolite Transporter (DMT) Superfamily	-	drug/metabolite
sequence01_3312409_3312726	DMT	The Drug/Metabolite Transporter (DMT) Superfamily	SMR	multidrug efflux (SMR)
sequence01_3361586_3362515	DMT	The Drug/Metabolite Transporter (DMT) Superfamily	-	drug/metabolite
sequence01_3369465_3368500	DMT	The Drug/Metabolite Transporter (DMT) Superfamily	-	drug/metabolite
sequence01_4127084_4128049	DMT	The Drug/Metabolite Transporter (DMT) Superfamily	RarD	chloramphenicol (RarD homolog)
sequence01_4347840_4348691	DMT	The Drug/Metabolite Transporter (DMT) Superfamily	-	drug/metabolite
sequence01_4382398_4383249	DMT	The Drug/Metabolite Transporter (DMT) Superfamily	-	drug/metabolite
sequence01_608285_607398	DMT	The Drug/Metabolite Transporter (DMT) Superfamily	DME	drug/metabolite
sequence01_840769_841674	DMT	The Drug/Metabolite Transporter (DMT) Superfamily	-	drug/metabolite
sequence38_12036_12887	DMT	The Drug/Metabolite Transporter (DMT) Superfamily	-	drug/metabolite
sequence46_111754_112605	DMT	The Drug/Metabolite Transporter (DMT) Superfamily	-	drug/metabolite
sequence01_767301_769565	DNA-T	The Bacterial Competence-related DNA Transformation Transporter (DNA-T) Family	-	-
sequence01_3124652_3125854	ESS	The Glutamate:Na+ Symporter (ESS) Family		sodium ion:glutamate symporter
sequence01_1947492_1946173	EVE1-C	The Envelope Virus E1 Channel (EVE1-C) Family	-	-
sequence01_2972737_2973117	F-ATPase	The H+- or Na+-translocating F-type, V-type and A-type ATPase (F-ATPase) Superfamily	-	protons
sequence01_2973126_2973941	F-ATPase	The H+- or Na+-translocating F-type, V-type and A-type ATPase (F-ATPase) Superfamily	-	protons
sequence01_2973991_2974230	F-ATPase	The H+- or Na+-translocating F-type, V-type and A-type ATPase (F-ATPase) Superfamily	-	protons
sequence01_2974279_2974749	F-ATPase	The H+- or Na+-translocating F-type, V-type and A-type ATPase (F-ATPase) Superfamily	-	protons

sequence01_2974764_2975297	F-ATPase	The H+- or Na+-translocating F-type, V-type and A-type ATPase (F-ATPase) Superfamily	-	protons
sequence01_2975310_2976851	F-ATPase	The H+- or Na+-translocating F-type, V-type and A-type ATPase (F-ATPase) Superfamily	-	protons
sequence01_2976903_2977766	F-ATPase	The H+- or Na+-translocating F-type, V-type and A-type ATPase (F-ATPase) Superfamily	-	protons
sequence01_2977798_2979180	F-ATPase	The H+- or Na+-translocating F-type, V-type and A-type ATPase (F-ATPase) Superfamily	-	protons
sequence01_2979201_2979620	F-ATPase	The H+- or Na+-translocating F-type, V-type and A-type ATPase (F-ATPase) Superfamily	-	protons
sequence01_2742484_2744802	FeoB	The Ferrous Iron Uptake (FeoB) Family	-	ferrous ion
sequence01_3308133_3307672	FeoB	The Ferrous Iron Uptake (FeoB) Family	-	ferrous ion
sequence01_2436289_2437218	FNT	The Formate-Nitrite Transporter (FNT) Family	-	formate/nitrite
sequence01_758386_757529	FNT	The Formate-Nitrite Transporter (FNT) Family	-	formate/nitrite
sequence01_2747467_2748783	GntP	The Gluconate:H+ Symporter (GntP) Family	-	gluconate
sequence01_2774409_2773069	GntP	The Gluconate:H+ Symporter (GntP) Family	-	gluconate
sequence01_3035592_3034255	GntP	The Gluconate:H+ Symporter (GntP) Family	-	gluconate
sequence01_1056497_1057897	GPH	The Glycoside-Pentoside-Hexuronide (GPH):Cation Symporter Family	-	multidrug efflux
sequence01_1318178_1319716	GPH	The Glycoside-Pentoside-Hexuronide (GPH):Cation Symporter Family	-	multidrug efflux
sequence01_1965511_1963928	GPH	The Glycoside-Pentoside-Hexuronide (GPH):Cation Symporter Family	-	multidrug efflux
sequence01_2931766_2933148	GPH	The Glycoside-Pentoside-Hexuronide (GPH):Cation Symporter Family	-	multidrug efflux
sequence01_2933192_2934616	GPH	The Glycoside-Pentoside-Hexuronide (GPH):Cation Symporter Family	-	multidrug efflux
sequence01_3117386_3118762	GPH	The Glycoside-Pentoside-Hexuronide (GPH):Cation Symporter Family	-	multidrug efflux
sequence01_3378968_3377424	GPH	The Glycoside-Pentoside-Hexuronide (GPH):Cation Symporter Family	-	multidrug efflux
sequence01_3445964_3447346	GPH	The Glycoside-Pentoside-Hexuronide (GPH):Cation Symporter Family	-	multidrug efflux
sequence01_3664322_3662925	GPH	The Glycoside-Pentoside-Hexuronide (GPH):Cation Symporter Family	-	multidrug efflux
sequence01_39125_37725	GPH	The Glycoside-Pentoside-Hexuronide (GPH):Cation Symporter Family	-	multidrug efflux

sequence01_977735_976404	GPH	The Glycoside-Pentoside-Hexuronide (GPH):Cation Symporter Family	-	multidrug efflux
sequence01_2466929_2467186	GPTS	General PTS	HPr	-
sequence01_2467232_2468959	GPTS	General PTS	EnzymeI	-
sequence01_4111920_4111648	GPTS	General PTS	HPr	-
sequence01_428115_425620	GPTS	General PTS	EnzymeI	-
sequence01_4586702_4588948	GPTS	General PTS	EnzymeI	-
sequence01_1333941_1332676	HAAAP	The Hydroxy/Aromatic Amino Acid Permease (HAAAP) Family	-	serine
sequence01_2003961_2005103	HAAAP	The Hydroxy/Aromatic Amino Acid Permease (HAAAP) Family	-	aromatic amino acid
sequence01_4153560_4154804	HAAAP	The Hydroxy/Aromatic Amino Acid Permease (HAAAP) Family	-	aromatic amino acid
sequence01_4189112_4190443	HAAAP	The Hydroxy/Aromatic Amino Acid Permease (HAAAP) Family	-	serine
sequence01_4194267_4195598	HAAAP	The Hydroxy/Aromatic Amino Acid Permease (HAAAP) Family	-	serine
sequence01_4554464_4553235	HAAAP	The Hydroxy/Aromatic Amino Acid Permease (HAAAP) Family	-	serine
sequence01_4618465_4617176	HAAAP	The Hydroxy/Aromatic Amino Acid Permease (HAAAP) Family	-	serine
sequence01_3377230_3375893	HCC	The HlyC/CorC (HCC) Family	-	hemolysin C (HlyC) homolog
sequence01_3836462_3835221	HCC	The HlyC/CorC (HCC) Family	-	heavy metal ion
sequence01_1968242_1966164	IIISP	The Type III (Virulence-related) Secretory Pathway (IIISP) Family	-	-
sequence01_1969383_1968235	IIISP	The Type III (Virulence-related) Secretory Pathway (IIISP) Family	-	-
sequence01_2063524_2065206	IIISP	The Type III (Virulence-related) Secretory Pathway (IIISP) Family	-	-
sequence01_2071936_2072310	IIISP	The Type III (Virulence-related) Secretory Pathway (IIISP) Family	-	-
sequence01_2072310_2073047	IIISP	The Type III (Virulence-related) Secretory Pathway (IIISP) Family	-	-
sequence01_2073058_2073327	IIISP	The Type III (Virulence-related) Secretory Pathway (IIISP) Family	-	-
sequence01_2073334_2074119	IIISP	The Type III (Virulence-related) Secretory Pathway (IIISP) Family	-	-

sequence01_857309_858142	ILT	The Iron/Lead Transporter (ILT) Superfamily	-	iron ion
sequence01_859314_860597	ILT	The Iron/Lead Transporter (ILT) Superfamily	-	iron ion
sequence38_64516_62432	IVSP	The Type IV (Conjugal DNA-Protein Transfer or VirB) Secretory Pathway (IVSP) Family	-	-
sequence39_11428_12009	IVSP	The Type IV (Conjugal DNA-Protein Transfer or VirB) Secretory Pathway (IVSP) Family	-	-
sequence39_12758_14161	IVSP	The Type IV (Conjugal DNA-Protein Transfer or VirB) Secretory Pathway (IVSP) Family	-	-
sequence39_30340_33738	IVSP	The Type IV (Conjugal DNA-Protein Transfer or VirB) Secretory Pathway (IVSP) Family	-	-
sequence39_35795_38350	IVSP	The Type IV (Conjugal DNA-Protein Transfer or VirB) Secretory Pathway (IVSP) Family	-	-
sequence46_31434_30376	IVSP	The Type IV (Conjugal DNA-Protein Transfer or VirB) Secretory Pathway (IVSP) Family	-	-
sequence46_57125_55770	IVSP	The Type IV (Conjugal DNA-Protein Transfer or VirB) Secretory Pathway (IVSP) Family	-	-
sequence01_4010741_4009749	KDGT	The 2-Keto-3-Deoxygluconate Transporter (KDGT) Family	-	2-keto-3-deoxygluconate
sequence01_2963819_2961951	KUP	The K+ Uptake Permease (KUP) Family	-	potassium ion uptake
sequence01_18493_16838	LctP	The Lactate Permease (LctP) Family	-	L-lactate
sequence01_2906869_2907528	LIV-E	The Branched Chain Amino Acid Exporter (LIV-E) Family	AzlC	branched-chain amino acid efflux (AzlC)
sequence01_168657_169976	LIVCS	The Branched Chain Amino Acid:Cation Symporter (LIVCS) Family	-	branched-chain amino acid
sequence01_4500676_4501311	LysE	The L-Lysine Exporter (LysE) Family	-	lysine efflux
sequence01_1111142_1109943	MFS	The Major Facilitator Superfamily (MFS)	-	multidrug efflux
sequence01_1113757_1114923	MFS	The Major Facilitator Superfamily (MFS)	-	multidrug efflux
sequence01_1247023_1245770	MFS	The Major Facilitator Superfamily (MFS)	-	multidrug efflux
sequence01_1257331_1256147	MFS	The Major Facilitator Superfamily (MFS)	-	multidrug efflux
sequence01_1315453_1316754	MFS	The Major Facilitator Superfamily (MFS)	-	glycerol-3-phosphate
sequence01_1390378_1388726	MFS	The Major Facilitator Superfamily (MFS)	-	multidrug efflux (EmrB/QacA subfamily)
sequence01_1461734_1460517	MFS	The Major Facilitator Superfamily (MFS)	-	multidrug efflux
sequence01_1466304_1465108	MFS	The Major Facilitator Superfamily (MFS)	-	multidrug efflux
sequence01_14703_13363	MFS	The Major Facilitator Superfamily (MFS)	-	metabolite (benzoate)

sequence01_152580_153794	MFS	The Major Facilitator Superfamily (MFS)	-	multidrug efflux
sequence01_1553724_1555103	MFS	The Major Facilitator Superfamily (MFS)	-	multidrug efflux (EmrB/QacA subfamily)
sequence01_1608353_1607178	MFS	The Major Facilitator Superfamily (MFS)	-	multidrug efflux
sequence01_1618925_1617435	MFS	The Major Facilitator Superfamily (MFS)	-	multidrug efflux (EmrB/QacA subfamily)
sequence01_1619828_1621213	MFS	The Major Facilitator Superfamily (MFS)	-	nitrate
sequence01_1797396_1795999	MFS	The Major Facilitator Superfamily (MFS)	-	nitrate
sequence01_1916506_1917879	MFS	The Major Facilitator Superfamily (MFS)	-	multidrug efflux (EmrB/QacA subfamily)
sequence01_2000046_2001464	MFS	The Major Facilitator Superfamily (MFS)	-	multidrug efflux (EmrB/QacA subfamily)
sequence01_204860_203499	MFS	The Major Facilitator Superfamily (MFS)	-	multidrug efflux
sequence01_2098438_2099739	MFS	The Major Facilitator Superfamily (MFS)	-	multidrug efflux
sequence01_2103675_2102476	MFS	The Major Facilitator Superfamily (MFS)	-	multidrug efflux
sequence01_2133727_2135043	MFS	The Major Facilitator Superfamily (MFS)	-	sugar
sequence01_2209647_2211062	MFS	The Major Facilitator Superfamily (MFS)	-	multidrug efflux (EmrB/QacA subfamily)
sequence01_2225433_2224363	MFS	The Major Facilitator Superfamily (MFS)	-	metabolite (alpha-ketoglutarate)
sequence01_2225612_2226712	MFS	The Major Facilitator Superfamily (MFS)	-	metabolite (alpha-ketoglutarate)
sequence01_222630_221155	MFS	The Major Facilitator Superfamily (MFS)	-	Acetyl-CoA:CoA antiporter
sequence01_2228068_2229345	MFS	The Major Facilitator Superfamily (MFS)	-	sugar
sequence01_2278015_2276867	MFS	The Major Facilitator Superfamily (MFS)	-	multidrug efflux
sequence01_2290023_2291255	MFS	The Major Facilitator Superfamily (MFS)	-	multidrug efflux
sequence01_2307170_2305971	MFS	The Major Facilitator Superfamily (MFS)	-	multidrug efflux
sequence01_2342480_2341128	MFS	The Major Facilitator Superfamily (MFS)	-	glycerol-3-phosphate
sequence01_2379067_2378627	MFS	The Major Facilitator Superfamily (MFS)	-	multidrug efflux
sequence01_2420061_2418883	MFS	The Major Facilitator Superfamily (MFS)	-	multidrug efflux
sequence01_2595349_2594210	MFS	The Major Facilitator Superfamily (MFS)	-	multidrug efflux
sequence01_2649505_2648198	MFS	The Major Facilitator Superfamily (MFS)	-	multidrug efflux
sequence01_2708682_2709866	MFS	The Major Facilitator Superfamily (MFS)	-	sugar efflux
sequence01_284159_282939	MFS	The Major Facilitator Superfamily (MFS)	-	multidrug efflux
sequence01_2854064_2855386	MFS	The Major Facilitator Superfamily (MFS)	-	glycerol-3-phosphate
sequence01_2902930_2901653	MFS	The Major Facilitator Superfamily (MFS)	-	glycerol-3-phosphate

sequence01_2954483_2955916	MFS	The Major Facilitator Superfamily (MFS)	-	multidrug efflux (EmrB/QacA subfamily)
sequence01_2993981_2992767	MFS	The Major Facilitator Superfamily (MFS)	-	multidrug efflux
sequence01_3011859_3013151	MFS	The Major Facilitator Superfamily (MFS)	-	D-galactonate
sequence01_3038983_3037799	MFS	The Major Facilitator Superfamily (MFS)	-	multidrug efflux
sequence01_3047276_3048586	MFS	The Major Facilitator Superfamily (MFS)	-	multidrug efflux
sequence01_3048719_3050110	MFS	The Major Facilitator Superfamily (MFS)	-	multidrug efflux
sequence01_3053513_3054703	MFS	The Major Facilitator Superfamily (MFS)	-	multidrug efflux
sequence01_3063490_3062333	MFS	The Major Facilitator Superfamily (MFS)	-	multidrug efflux
sequence01_3071195_3072679	MFS	The Major Facilitator Superfamily (MFS)	-	multidrug efflux (EmrB/QacA subfamily)
sequence01_3242604_3241324	MFS	The Major Facilitator Superfamily (MFS)	-	D-galactonate
sequence01_3250776_3251978	MFS	The Major Facilitator Superfamily (MFS)	-	oxalate:formate antiporter
sequence01_328117_329352	MFS	The Major Facilitator Superfamily (MFS)	-	sugar
sequence01_3347019_3345745	MFS	The Major Facilitator Superfamily (MFS)	-	multidrug efflux
sequence01_335324_333888	MFS	The Major Facilitator Superfamily (MFS)	-	multidrug efflux (EmrB/QacA subfamily)
sequence01_3495919_3494576	MFS	The Major Facilitator Superfamily (MFS)	-	4-hydroxyphenylacetate
sequence01_3559891_3561423	MFS	The Major Facilitator Superfamily (MFS)	-	multidrug efflux
sequence01_3619167_3620345	MFS	The Major Facilitator Superfamily (MFS)	-	multidrug efflux
sequence01_3777287_3775740	MFS	The Major Facilitator Superfamily (MFS)	-	multidrug efflux (EmrB/QacA subfamily)
sequence01_3780636_3779452	MFS	The Major Facilitator Superfamily (MFS)	-	multidrug efflux
sequence01_392102_393328	MFS	The Major Facilitator Superfamily (MFS)	-	multidrug efflux
sequence01_4011388_4012890	MFS	The Major Facilitator Superfamily (MFS)	-	multidrug efflux
sequence01_4081820_4083310	MFS	The Major Facilitator Superfamily (MFS)	-	multidrug efflux
sequence01_4206125_4204827	MFS	The Major Facilitator Superfamily (MFS)	-	D-galactonate
sequence01_4286437_4284971	MFS	The Major Facilitator Superfamily (MFS)	-	multidrug efflux
sequence01_4323065_4324330	MFS	The Major Facilitator Superfamily (MFS)	-	multidrug efflux
sequence01_4449324_4448068	MFS	The Major Facilitator Superfamily (MFS)	-	multidrug efflux
sequence01_4464499_4463102	MFS	The Major Facilitator Superfamily (MFS)	-	sugar
sequence01_4471341_4470058	MFS	The Major Facilitator Superfamily (MFS)	-	multidrug efflux
sequence01_4477111_4478367	MFS	The Major Facilitator Superfamily (MFS)	-	D-galactonate

sequence01_45273_43954	MFS	The Major Facilitator Superfamily (MFS)	-	D-galactonate
sequence01_4561942_4563357	MFS	The Major Facilitator Superfamily (MFS)	-	sugar
sequence01_4581976_4583169	MFS	The Major Facilitator Superfamily (MFS)	-	multidrug efflux
sequence01_4624426_4625781	MFS	The Major Facilitator Superfamily (MFS)	-	D-galactonate
sequence01_4645698_4644301	MFS	The Major Facilitator Superfamily (MFS)	-	glycerol-3-phosphate
sequence01_4668429_4667227	MFS	The Major Facilitator Superfamily (MFS)	-	multidrug efflux
sequence01_4725723_4727021	MFS	The Major Facilitator Superfamily (MFS)	-	multidrug efflux
sequence01_580546_579584	MFS	The Major Facilitator Superfamily (MFS)	-	multidrug efflux
sequence01_614210_615781	MFS	The Major Facilitator Superfamily (MFS)	-	multidrug efflux (EmrB/QacA subfamily)
sequence01_643675_642374	MFS	The Major Facilitator Superfamily (MFS)	-	D-galactonate
sequence01_667910_669142	MFS	The Major Facilitator Superfamily (MFS)	-	multidrug efflux
sequence01_671271_670057	MFS	The Major Facilitator Superfamily (MFS)	-	multidrug efflux
sequence01_752422_753570	MFS	The Major Facilitator Superfamily (MFS)	-	multidrug efflux
sequence01_77353_78528	MFS	The Major Facilitator Superfamily (MFS)	-	multidrug efflux
sequence01_883445_882210	MFS	The Major Facilitator Superfamily (MFS)	-	multidrug efflux
sequence01_892482_891274	MFS	The Major Facilitator Superfamily (MFS)	-	multidrug efflux
sequence01_909392_908181	MFS	The Major Facilitator Superfamily (MFS)	-	multidrug efflux
sequence01_972924_971737	MFS	The Major Facilitator Superfamily (MFS)	-	cyanate
sequence33_2348_3547	MFS	The Major Facilitator Superfamily (MFS)	-	multidrug efflux
sequence41_8691_9656	MFS	The Major Facilitator Superfamily (MFS)	-	metabolite (alpha-ketoglutarate)
sequence41_968_3	MFS	The Major Facilitator Superfamily (MFS)	-	metabolite (alpha-ketoglutarate)
sequence01_2319271_2317835	MgtE	The Mg2+ Transporter-E (MgtE) Family	-	magnesium ion
sequence01_835543_834542	MgtE	The Mg2+ Transporter-E (MgtE) Family	-	magnesium ion
sequence01_3866689_3865844	MIP	The Major Intrinsic Protein (MIP) Family	-	glycerol uptake
sequence01_723870_723073	MIP	The Major Intrinsic Protein (MIP) Family	-	glycerol uptake
sequence01_1683137_1682154	MIT	The CorA Metal Ion Transporter (MIT) Family	-	magnesium/cobalt ion
sequence01_3211850_3212800	MIT	The CorA Metal Ion Transporter (MIT) Family	-	magnesium/cobalt ion
sequence01_1107585_1106224	MOP	The Multidrug/Oligosaccharidyl-lipid/Polysaccharide (MOP) Flippase Superfamily	MATE	multidrug efflux

sequence01_2138292_2136874	MOP	The Multidrug/Oligosaccharidyl-lipid/Polysaccharide (MOP) Flippase Superfamily	MATE	multidrug efflux
sequence01_2164178_2162958	MOP	The Multidrug/Oligosaccharidyl-lipid/Polysaccharide (MOP) Flippase Superfamily	-	multidrug efflux
sequence01_2173283_2171805	MOP	The Multidrug/Oligosaccharidyl-lipid/Polysaccharide (MOP) Flippase Superfamily	PST	polysaccharide export
sequence01_3191092_3192342	MOP	The Multidrug/Oligosaccharidyl-lipid/Polysaccharide (MOP) Flippase Superfamily	PST	polysaccharide export
sequence01_3938537_3939874	MOP	The Multidrug/Oligosaccharidyl-lipid/Polysaccharide (MOP) Flippase Superfamily	MATE	multidrug efflux
sequence01_895019_896554	MOP	The Multidrug/Oligosaccharidyl-lipid/Polysaccharide (MOP) Flippase Superfamily	MVF	virulence factor MviN
sequence01_1987286_1986399	Mot/Exb	The H+- or Na+-translocating Bacterial Flagellar Motor 1ExbBD Outer Membrane Transport Energizer (Mo	-	-
sequence01_4411388_4412119	Mot/Exb	The H+- or Na+-translocating Bacterial Flagellar Motor 1ExbBD Outer Membrane Transport Energizer (Mo	-	-
sequence01_4412124_4412549	Mot/Exb	The H+- or Na+-translocating Bacterial Flagellar Motor 1ExbBD Outer Membrane Transport Energizer (Mo	-	-
sequence01_534386_535078	Mot/Exb	The H+- or Na+-translocating Bacterial Flagellar Motor 1ExbBD Outer Membrane Transport Energizer (Mo	-	-
sequence01_535082_535510	Mot/Exb	The H+- or Na+-translocating Bacterial Flagellar Motor 1ExbBD Outer Membrane Transport Energizer (Mo	-	-
sequence01_3452375_3453097	MPT	The Mitochondrial Protein Translocase (MPT) Family	-	-
sequence01_2659934_2660347	MscL	The Large Conductance Mechanosensitive Ion Channel (MscL) Family	-	large-conductance mechanosensitive ion channel
sequence01_1211603_1212712	MscS	The Small Conductance Mechanosensitive Ion Channel (MscS) Family	-	small-conductance mechanosensitive ion channel
sequence01_267430_270777	MscS	The Small Conductance Mechanosensitive Ion Channel (MscS) Family	-	small-conductance mechanosensitive ion channel
sequence01_3325213_3321890	MscS	The Small Conductance Mechanosensitive Ion Channel (MscS) Family	-	small-conductance mechanosensitive ion channel
sequence01_333820_332576	MscS	The Small Conductance Mechanosensitive Ion Channel (MscS) Family	-	small-conductance mechanosensitive ion channel

sequence01_4498516_4499370	MscS	The Small Conductance Mechanosensitive Ion Channel (MscS) Family	-	small-conductance mechanosensitive ion channel
sequence01_603898_601688	MscS	The Small Conductance Mechanosensitive Ion Channel (MscS) Family	-	small-conductance mechanosensitive ion channel
sequence01_831986_833599	MscS	The Small Conductance Mechanosensitive Ion Channel (MscS) Family	-	small-conductance mechanosensitive ion channel
sequence01_2675559_2676032	MTB	The Outer Membrane Protein Secreting Main Terminal Branch (MTB)	-	-
sequence01_3657556_3656372	MTB	The Outer Membrane Protein Secreting Main Terminal Branch (MTB)	-	-
sequence01_3659375_3658938	MTB	The Outer Membrane Protein Secreting Main Terminal Branch (MTB)	-	-
sequence01_697250_698023	MTB	The Outer Membrane Protein Secreting Main Terminal Branch (MTB)	-	-
sequence01_701446_702654	MTB	The Outer Membrane Protein Secreting Main Terminal Branch (MTB)	-	-
sequence01_702658_703107	MTB	The Outer Membrane Protein Secreting Main Terminal Branch (MTB)	-	-
sequence01_703107_703592	MTB	The Outer Membrane Protein Secreting Main Terminal Branch (MTB)	-	-
sequence01_703585_703965	MTB	The Outer Membrane Protein Secreting Main Terminal Branch (MTB)	-	-
sequence01_703962_704570	MTB	The Outer Membrane Protein Secreting Main Terminal Branch (MTB)	-	-
sequence01_704567_705577	MTB	The Outer Membrane Protein Secreting Main Terminal Branch (MTB)	-	-
sequence01_706723_707208	MTB	The Outer Membrane Protein Secreting Main Terminal Branch (MTB)	-	-
sequence01_707205_708002	MTB	The Outer Membrane Protein Secreting Main Terminal Branch (MTB)	-	-
sequence01_4087360_4086110	NCS1	The Nucleobase:Cation Symporter-1 (NCS1) Family	-	cytosine/purines/uracil/thiamine/allantoin
sequence01_2524691_2523402	NCS2	The Nucleobase:Cation Symporter-2 (NCS2) Family	-	xanthine/uracil
sequence01_2988926_2990266	NCS2	The Nucleobase:Cation Symporter-2 (NCS2) Family	-	xanthine/uracil
sequence01_3124426_3123035	NCS2	The Nucleobase:Cation Symporter-2 (NCS2) Family	-	xanthine/uracil
sequence01_3961054_3962403	NCS2	The Nucleobase:Cation Symporter-2 (NCS2) Family	-	xanthine/uracil
sequence01_843926_842604	NCS2	The Nucleobase:Cation Symporter-2 (NCS2) Family	-	xanthine/uracil
sequence01_3565391_3566566	NhaA	The NhaA Na+:H+ Antiporter (NhaA) Family	-	sodium ion:proton antiporter

sequence01_1886487_1888025	NhaB	The NhaB Na+:H+ Antiporter (NhaB) Family	-	sodium ion:proton antiporter
sequence01_254854_254039	NiCoT	The Ni2+-Co2+ Transporter (NiCoT) Family	-	nickel ion
sequence01_2592177_2591197	NiCoT	The Ni2+-Co2+ Transporter (NiCoT) Family	-	nickel ion
sequence01_4013877_4012924	NiCoT	The Ni2+-Co2+ Transporter (NiCoT) Family	-	nickel ion
sequence01_4696735_4697757	NiCoT	The Ni2+-Co2+ Transporter (NiCoT) Family	-	nickel ion
sequence01_4727409_4728539	NiCoT	The Ni2+-Co2+ Transporter (NiCoT) Family	-	nickel ion
sequence01_2451533_2450295	Nramp	The Metal Ion (Mn2+-iron) Transporter (Nramp) Family	-	manganese/iron ion
sequence01_588062_587067	OMF	The Outer Membrane Factor (OMF) Family	-	-
sequence01_1865669_1864431	OOP	The OmpA-OmpF Porin (OOP) Family	-	-
sequence01_1986402_1985473	OOP	The OmpA-OmpF Porin (OOP) Family	-	-
sequence01_2997772_2996129	Oxa1	The Cytochrome Oxidase Biogenesis (Oxa1) Family	-	60 KD inner membrane protein OxaA homolog
sequence01_1518386_1515945	P-ATPase	The P-type ATPase (P-ATPase) Superfamily	-	zinc/cadmium/cobalt ion
sequence01_2126837_2124144	P-ATPase	The P-type ATPase (P-ATPase) Superfamily	-	calcium ion/manganese ion
sequence01_2801441_2803612	P-ATPase	The P-type ATPase (P-ATPase) Superfamily	-	zinc/cadmium/cobalt ion
sequence01_290087_287589	P-ATPase	The P-type ATPase (P-ATPase) Superfamily	-	copper ion
sequence01_3427138_3429846	P-ATPase	The P-type ATPase (P-ATPase) Superfamily	-	magnesium ion
sequence01_345132_347840	P-ATPase	The P-type ATPase (P-ATPase) Superfamily	-	calcium ion/manganese ion
sequence01_502539_501964	P-ATPase	The P-type ATPase (P-ATPase) Superfamily	-	potassium ion
sequence01_504600_502552	P-ATPase	The P-type ATPase (P-ATPase) Superfamily	-	potassium ion
sequence01_506298_504619	P-ATPase	The P-type ATPase (P-ATPase) Superfamily	-	potassium ion
sequence01_4555045_4554581	PACE	The Chlorhexadine Exporter (CHX) family	-	chlorhexidine
sequence01_2837126_2838625	PiT	The Inorganic Phosphate Transporter (PiT) Family	-	phosphate
sequence01_3912696_3914327	PNaS	The Phosphate:Na+ Symporter (PNaS) Family	-	sodium ion:phosphate symporter
sequence01_1136746_1135238	POT	The Proton-dependent Oligopeptide Transporter (POT) Family	-	proton:dipeptide/tripeptide symporter
sequence01_2555066_2553525	POT	The Proton-dependent Oligopeptide Transporter (POT) Family	-	proton:dipeptide/tripeptide symporter
sequence01_147420_148640	PUP	The Putative Peptide Uptake or Activated Fatty Acid Export Permease (PUP) Family	-	peptide uptake

sequence01_1464205_1464870	RhtB	The Resistance to Homoserine/Threonine (RhtB) Family	-	neutral amino acid/multiple antibiotic resistance
sequence01_1767999_1767364	RhtB	The Resistance to Homoserine/Threonine (RhtB) Family	-	amino acid efflux
sequence01_3217311_3217931	RhtB	The Resistance to Homoserine/Threonine (RhtB) Family	-	amino acid efflux
sequence01_3218548_3217928	RhtB	The Resistance to Homoserine/Threonine (RhtB) Family	-	amino acid efflux
sequence01_3296033_3296623	RhtB	The Resistance to Homoserine/Threonine (RhtB) Family	-	amino acid efflux
sequence01_3482577_3481939	RhtB	The Resistance to Homoserine/Threonine (RhtB) Family	-	amino acid efflux
sequence01_1126665_1125811	RND	The Resistance-Nodulation-Cell Division (RND) Superfamily	-	multidrug efflux
sequence01_1388703_1387621	RND	The Resistance-Nodulation-Cell Division (RND) Superfamily	-	multidrug efflux
sequence01_1522186_1519040	RND	The Resistance-Nodulation-Cell Division (RND) Superfamily	HME	cobalt/zinc/cadmium ion efflux (HME subfamily)
sequence01_181610_183424	RND	The Resistance-Nodulation-Cell Division (RND) Superfamily	SecDF	protein export (SecDF)
sequence01_2128874_2131936	RND	The Resistance-Nodulation-Cell Division (RND) Superfamily	HAE1	multidrug/solvent efflux (HAE1 subfamily)
sequence01_2203446_2206568	RND	The Resistance-Nodulation-Cell Division (RND) Superfamily	HAE1	multidrug/solvent efflux (HAE1 subfamily)
sequence01_2206569_2209646	RND	The Resistance-Nodulation-Cell Division (RND) Superfamily	HAE1	multidrug/solvent efflux (HAE1 subfamily)
sequence01_2501534_2504647	RND	The Resistance-Nodulation-Cell Division (RND) Superfamily	HAE1	multidrug/solvent efflux (HAE1 subfamily)
sequence01_2544095_2540943	RND	The Resistance-Nodulation-Cell Division (RND) Superfamily	HAE1	multidrug/solvent efflux (HAE1 subfamily)
sequence01_265305_262159	RND	The Resistance-Nodulation-Cell Division (RND) Superfamily	HAE1	multidrug/solvent efflux (HAE1 subfamily)
sequence01_2816715_2819036	RND	The Resistance-Nodulation-Cell Division (RND) Superfamily	-	multidrug efflux
sequence01_3068014_3064904	RND	The Resistance-Nodulation-Cell Division (RND) Superfamily	HAE1	multidrug/solvent efflux (HAE1 subfamily)
sequence01_349021_352176	RND	The Resistance-Nodulation-Cell Division (RND) Superfamily	HAE1	multidrug/solvent efflux (HAE1 subfamily)

sequence01_4018768_4015655	RND	The Resistance-Nodulation-Cell Division (RND) Superfamily	HAE1	multidrug/solvent efflux (HAE1 subfamily)
sequence01_4065494_4066426	RND	The Resistance-Nodulation-Cell Division (RND) Superfamily	-	multidrug efflux
sequence01_437871_440987	RND	The Resistance-Nodulation-Cell Division (RND) Superfamily	HME	cobalt/zinc/cadmium ion efflux (HME subfamily)
sequence01_613119_614213	RND	The Resistance-Nodulation-Cell Division (RND) Superfamily	-	multidrug efflux
sequence01_1015643_1015960	SSPTS	Sugar Specific PTS	EnzymeIIB	cellobiose
sequence01_1016041_1017399	SSPTS	Sugar Specific PTS	EnzymeIIABC	cellobiose
sequence01_1017447_1017794	SSPTS	Sugar Specific PTS	EnzymeIIA	cellobiose
sequence01_1149762_1148365	SSPTS	Sugar Specific PTS	EnzymeIIABC	glucose/maltose/N-acetylglucosamine
sequence01_1191341_1190973	SSPTS	Sugar Specific PTS	EnzymeIIBC	glucose/maltose/N-acetylglucosamine
sequence01_1582188_1580818	SSPTS	Sugar Specific PTS	EnzymeIIABC	glucose/maltose/N-acetylglucosamine
sequence01_1906563_1907525	SSPTS	Sugar Specific PTS	EnzymeIIAB	mannose/fructose
sequence01_1907573_1908373	SSPTS	Sugar Specific PTS	EnzymeIIC	mannose/fructose
sequence01_1908386_1909237	SSPTS	Sugar Specific PTS	EnzymeIID	mannose/fructose
sequence01_201081_199813	SSPTS	Sugar Specific PTS	EnzymeIIABC	cellobiose
sequence01_2287630_2285945	SSPTS	Sugar Specific PTS	EnzymeIIBC	fructose
sequence01_2289715_2288585	SSPTS	Sugar Specific PTS	EnzymeIIA	mannitol/fructose
sequence01_2394244_2392853	SSPTS	Sugar Specific PTS	EnzymeIIC	ascorbate
sequence01_2394538_2394266	SSPTS	Sugar Specific PTS	EnzymeIIB	galactitol
sequence01_2395061_2394618	SSPTS	Sugar Specific PTS	EnzymeIIA	fructose
sequence01_2469000_2469509	SSPTS	Sugar Specific PTS	EnzymeIIABC	glucose
sequence01_2518820_2520139	SSPTS	Sugar Specific PTS	EnzymeIIC	cellobiose
sequence01_2576755_2578299	SSPTS	Sugar Specific PTS	EnzymeIIABC	glucose/maltose/N-acetylglucosamine
sequence01_2611454_2610090	SSPTS	Sugar Specific PTS	EnzymeIIABC	glucose/maltose/N-acetylglucosamine
sequence01_28665_26758	SSPTS	Sugar Specific PTS	EnzymeIIABC	mannitol
sequence01_3019719_3019396	SSPTS	Sugar Specific PTS	EnzymeIIB	galactitol
sequence01_3021073_3019748	SSPTS	Sugar Specific PTS	EnzymeIIC	ascorbate
sequence01_3023922_3025544	SSPTS	Sugar Specific PTS	EnzymeIIABC	glucose/maltose/N-acetylglucosamine

sequence01_3057613_3057308	SSPTS	Sugar Specific PTS	EnzymeIIA	cellobiose
sequence01_3060335_3059010	SSPTS	Sugar Specific PTS	EnzymeIIABC	cellobiose
sequence01_3060661_3060347	SSPTS	Sugar Specific PTS	EnzymeIIB	cellobiose
sequence01_310388_308889	SSPTS	Sugar Specific PTS	EnzymeIIABC	glucose/maltose/N-acetylglucosamine
sequence01_3277403_3277086	SSPTS	Sugar Specific PTS	EnzymeIIA	cellobiose
sequence01_3277711_3277403	SSPTS	Sugar Specific PTS	EnzymeIIB	cellobiose
sequence01_3354836_3356236	SSPTS	Sugar Specific PTS	EnzymeIIC	ascorbate
sequence01_3356249_3356554	SSPTS	Sugar Specific PTS	EnzymeIIB	galactitol
sequence01_3356564_3357031	SSPTS	Sugar Specific PTS	EnzymeIIA	fructose
sequence01_3416815_3416243	SSPTS	Sugar Specific PTS	EnzymeIIA	fructose
sequence01_3420828_3419425	SSPTS	Sugar Specific PTS	EnzymeIIBC	fructose
sequence01_3425676_3424258	SSPTS	Sugar Specific PTS	EnzymeIIABC	glucose/maltose/N-acetylglucosamine
sequence01_3514237_3515583	SSPTS	Sugar Specific PTS	EnzymeIIABC	cellobiose
sequence01_3576302_3575484	SSPTS	Sugar Specific PTS	EnzymeIID	mannose/fructose
sequence01_3577099_3576302	SSPTS	Sugar Specific PTS	EnzymeIIC	mannose/fructose
sequence01_3577604_3577110	SSPTS	Sugar Specific PTS	EnzymeIIB	mannose/fructose
sequence01_3578043_3577615	SSPTS	Sugar Specific PTS	EnzymeIIA	mannose/fructose
sequence01_3689091_3689531	SSPTS	Sugar Specific PTS	EnzymeIIA	mannose/fructose
sequence01_4113308_4112817	SSPTS	Sugar Specific PTS	EnzymeIIA	fructose
sequence01_4172550_4170979	SSPTS	Sugar Specific PTS	EnzymeIIABC	glucose/maltose/N-acetylglucosamine
sequence01_4176509_4176075	SSPTS	Sugar Specific PTS	EnzymeIIA	mannose/fructose
sequence01_4177405_4176527	SSPTS	Sugar Specific PTS	EnzymeIID	mannose/fructose
sequence01_4178174_4177395	SSPTS	Sugar Specific PTS	EnzymeIIC	mannose/fructose
sequence01_4178658_4178185	SSPTS	Sugar Specific PTS	EnzymeIIB	mannose/fructose
sequence01_431524_430274	SSPTS	Sugar Specific PTS	EnzymeIIC	fructose
sequence01_431875_431552	SSPTS	Sugar Specific PTS	EnzymeIIB	fructose
sequence01_4489279_4489722	SSPTS	Sugar Specific PTS	EnzymeIIA	fructose
sequence01_4489750_4491129	SSPTS	Sugar Specific PTS	EnzymeIIBC	mannitol
sequence01_4574144_4575469	SSPTS	Sugar Specific PTS	EnzymeIIC	cellobiose

sequence01_4641291_4642850	SSPTS	Sugar Specific PTS	EnzymeIIABC	glucose/maltose/N-acetylglucosamine
sequence01_4692604_4691153	SSPTS	Sugar Specific PTS	EnzymeIIABC	glucose/maltose/N-acetylglucosamine
sequence01_4705564_4705202	SSPTS	Sugar Specific PTS	EnzymeIIA	glucitol/sorbitol
sequence01_4706536_4705577	SSPTS	Sugar Specific PTS	EnzymeIIB	glucitol/sorbitol
sequence01_4707096_4706533	SSPTS	Sugar Specific PTS	EnzymeIIC	glucitol/sorbitol
sequence01_478564_480513	SSPTS	Sugar Specific PTS	EnzymeIIABC	glucose/maltose/N-acetylglucosamine
sequence01_525439_527355	SSPTS	Sugar Specific PTS	EnzymeIIABC	fructose
sequence01_635067_636389	SSPTS	Sugar Specific PTS	EnzymeIIBC	cellobiose
sequence01_926811_928244	SSPTS	Sugar Specific PTS	EnzymeIIABC	glucose/maltose/N-acetylglucosamine
sequence01_166952_168247	SSS	The Solute:Sodium Symporter (SSS) Family	-	sodium ion:proline symporter
sequence01_3027927_3029642	SSS	The Solute:Sodium Symporter (SSS) Family	-	sodium ion:proline symporter
sequence01_3968043_3966394	SSS	The Solute:Sodium Symporter (SSS) Family	-	sodium ion:proline symporter
sequence01_3984431_3986728	SSS	The Solute:Sodium Symporter (SSS) Family	-	sodium ion:proline symporter
sequence01_4045314_4043863	SSS	The Solute:Sodium Symporter (SSS) Family	-	sodium ion:panthothenate symporter
sequence01_4632507_4629748	SSS	The Solute:Sodium Symporter (SSS) Family	-	sodium ion:proline symporter
sequence01_501963_499276	SSS	The Solute:Sodium Symporter (SSS) Family	-	sodium ion:proline symporter
sequence01_686582_688051	SSS	The Solute:Sodium Symporter (SSS) Family	-	sodium ion:proline symporter
sequence01_853800_855308	SSS	The Solute:Sodium Symporter (SSS) Family	-	sodium ion:proline symporter
sequence01_1826090_1827769	SulP	The Sulfate Permease (SulP) Family	-	sulfate
sequence01_2316475_2317872	SulP	The Sulfate Permease (SulP) Family	-	sulfate
sequence31_14792_13314	SulP	The Sulfate Permease (SulP) Family	-	sulfate
sequence01_3231311_3231565	Tat	The Twin Arginine Targeting (Tat) Family	-	protein export
sequence01_3231569_3232111	Tat	The Twin Arginine Targeting (Tat) Family	-	protein export
sequence01_3232114_3232884	Tat	The Twin Arginine Targeting (Tat) Family	-	protein export
sequence01_443709_443912	Tat	The Twin Arginine Targeting (Tat) Family	-	protein export
sequence01_1439286_1440281	TDT	The Telurite-resistance/Dicarboxylate Transporter (TDT) Family	-	tellurite
sequence39_28319_28582	TDT	The Telurite-resistance/Dicarboxylate Transporter (TDT) Family	-	tellurite
sequence01_1906100_1904541	TerC	The Tellurium Ion Resistance (TerC) Family	-	tellurium ion efflux

sequence01_2191173_2192756	TerC	The Tellurium Ion Resistance (TerC) Family	-	tellurium ion efflux
sequence01_2835610_2834585	TerC	The Tellurium Ion Resistance (TerC) Family	-	tellurium ion efflux
sequence01_4212141_4211176	TerC	The Tellurium Ion Resistance (TerC) Family	-	tellurium ion efflux
sequence44_13804_14844	TerC	The Tellurium Ion Resistance (TerC) Family	-	tellurium ion efflux
sequence45_13804_14844	TerC	The Tellurium Ion Resistance (TerC) Family	-	tellurium ion efflux
sequence01_3511653_3510877	ThrE	The Threonine/Serine Exporter (ThrE) Family	-	-
sequence01_4055370_4055858	ThrE	The Threonine/Serine Exporter (ThrE) Family	-	-
sequence01_2804998_2803712	TRAP-T	The Tripartite ATP-independent Periplasmic Transporter (TRAP-T) Family	-	C4-dicarboxylate
sequence01_2805518_2805009	TRAP-T	The Tripartite ATP-independent Periplasmic Transporter (TRAP-T) Family	-	C4-dicarboxylate
sequence01_2806502_2805531	TRAP-T	The Tripartite ATP-independent Periplasmic Transporter (TRAP-T) Family	-	C4-dicarboxylate
sequence01_4500475_4499417	TRAP-T	The Tripartite ATP-independent Periplasmic Transporter (TRAP-T) Family	-	C4-dicarboxylate
sequence01_3142690_3142073	TRIC	The Homotrimeric Cation Channel (TRIC) Family	-	-
sequence01_3725593_3724970	TRIC	The Homotrimeric Cation Channel (TRIC) Family	-	-
sequence01_72898_74349	Trk	The K+ Transporter (Trk) Family	-	potassium ion uptake
sequence01_2350974_2351954	TTT	The Tricarboxylate Transporter (TTT) Family	TctC	tricarboxylate (TctC)
sequence01_2351966_2352397	TTT	The Tricarboxylate Transporter (TTT) Family	-	tricarboxylate
sequence01_2352409_2353923	TTT	The Tricarboxylate Transporter (TTT) Family	TctA	tricarboxylate (TctA)
sequence01_2460278_2461273	UBS1	The Unknown BART Superfamily-1 (UBS1) Family	-	sodium ion/
sequence01_1771740_1770904	VIC	The Voltage-gated Ion Channel (VIC) Superfamily	-	potassium ion channel
sequence01_4457200_4456634	YggT	The YggT or Fanciful K+ Uptake-B (FkuB; YggT) Family	-	-
sequence01_4303795_4303022	ZIP	The Zinc (Zn2+)-Iron (Fe2+) Permease (ZIP) Family	-	zinc ion
