# Supplementary material for: Genomic Landscape Highlights Molecular Mechanisms Involved in Silicate Solubilization, Stress Tolerance, and Potential Growth-Promoting Activity of Bacterium Enterobacter sp. LR6
Source: Cells. 2022 Nov 15;11(22):3622. doi: 10.3390/cells11223622 (PMC9688052; doi:10.3390/cells11223622)
Supplement: Supplementary file 1 [file cells-11-03622-s001.zip › cells-1917087-supplementary/cells-1917087-revised SM/Table S2.pdf]

Table S2. Presence of important genes and their location in LR6 genome.

S.No.	Protein	Gene	Location	Predicted Function
Plant growth promoting genes				
1.	Silicase	Carbonic anhydrase	sequence01_4042835_4042092	Helps to dissolve silicate
2.	Alkaline phosphatase	phoA	sequence01_2311656_2313416	Solubilize insoluble phosphate
3.	Phosphate starvation-inducible protein	phoH	sequence01_468363_467317	It enables cells to use limited phosphate resources more efficiently
4.	Phosphate regulon sensor protein,	PhoR	sequence01_166952_168247	It enables cells to use limited phosphate resources more efficiently
5.	Phosphate regulon transcriptional regulatory protein	PhoB	sequence01_166241_166930	It enables cells to use limited phosphate resources more efficiently
6.	pyruvate decarboxylase	pyruvate decarboxylase	sequence01_2448602_2446944	Production of indole-3-acetic acid
7.	PQQ synthesis protein	PqqF, PqqE, PqqD, PqqB	sequence01_1230452_1228176, sequence01_1231600_1230458, sequence01_1231865_1231587, sequence01_1233543_1232626	Mineral phosphate solubilization (MPS), bio-control efficacy, antioxidant activity
Genes responsible for motility/chemotaxis				

	Flagellar synthesis operons	flgNMABCDEFGHIJKL, flhEAB, cheZYBR, cheWA, motBA, flhCD, fliCDSTEFGHJKLMNOPQR, and fliEFHIJKLMNOPQR	sequence01_897039_896614, sequence01_861003_861119, sequence01_898090_897431, sequence01_898248_898664, sequence01_898668_899072, sequence01_899084_899794, sequence01_899821_901029, sequence01_901050_901805, sequence01_901817_902599, sequence01_902657_903355, sequence01_903368_904465, sequence01_904466_905419, sequence01_905495_907135, sequence01_907152_908105, sequence01_1966164_1965772, sequence01_1968242_1966164, sequence01_1969383_1968235, sequence01_1988341_1987991, sequence01_1970178_1969534, sequence01_1972508_1971642, sequence01_1971645_1970596, sequence01_1970578_1970189, sequence01_1983439_1982936, sequence01_46245_45319, sequence01_1986402_1985473, sequence01_1987286_1986399, sequence01_1987988_1987410, sequence01_1988341_1987991, sequence01_2024731_2023451, sequence01_2025001_2026413,	Motility

		sequence01_2026444_2026854, sequence01_2026854_2027225, sequence01_2063295_2062981, sequence01_2063524_2065206, sequence01_2065199_2066197, sequence01_2066190_2066897, sequence01_2066897_2068267, sequence01_2068289_2068732, sequence01_2068729_2069937, sequence01_2070044_2070514, sequence01_2070519_2071523, sequence01_2071520_2071933, sequence01_2071936_2072310, sequence01_2072310_2073047, sequence01_2073058_2073327, sequence01_2073334_2074119	
Putative chemotaxis protein	cheA	sequence01_46245_45319	chemotaxis
Methyl-accepting chemotaxis sensory transducer	mcp	sequence01_1470589_1472178,	chemotaxis
Methyl-accepting chemotaxis citrate transducer	tcp	sequence01_1223309_1221648, sequence01_1578271_1576721	chemotaxis

	Methyl-accepting chemotaxis protein I	tsr1, tsr2	sequence01_3457602_3458288	chemotaxis
	Methyl-accepting chemotaxis protein II	tarA	sequence01_1975841_1974174	chemotaxis
	Methyl-accepting chemotaxis protein III	trg	sequence01_1426997_1428688	chemotaxis
	Methyl-accepting chemotaxis protein IV	tap	sequence01_1974141_1972528	chemotaxis
	chemotaxis protein	cheZ, cheR, CheV	sequence01_1970178_1969534, sequence01_1972508_1971642, sequence01_2362889_2363893	chemotaxis
Colonization				
	Filamentous hemagglutinin-like adhesins	Filamentous hemagglutinin	sequence01_3410067_3411203	Adhesion of bacteria to the host surface
Antibiotic synthesis				
	antibiotic biosynthesis monooxygenase	antibiotic biosynthesis monooxygenase gene	sequence01_3476106_3476429	Plays a role in the biosynthesis of aromatic polyketides
